# Comparative Analysis of Gut Metabolites in *Meles leucurus* Across Different Seasons

**DOI:** 10.3390/ani16203182

**Published:** 2026-10-10

**Authors:** Xiaohan Ma, Yifan Zhang, Shuyuan Dong, Mengyao Sun, Ziyi Song, Xiuxiu Wang, Lei Chen

**Affiliations:** 1 School of Life Sciences, Qufu Normal University, Qufu 273165, China; 2 Shandong Key Laboratory of Wetland Ecology and Biodiversity Conservation in the Lower Yellow River, Qufu 273165, China

**Keywords:** *Meles leucurus*, gut metabolites, seasonal adaptation, untargeted metabolomics, LCMS

## Abstract

Seasonal changes in ambient temperature and food supply constantly challenge the nutritional homeostasis of wild mammals. Intestinal metabolites bridge host physiology, gut microbiota and external environments, and help uncover wildlife adaptive mechanisms. As an omnivorous burrowing hibernator, *Meles leucurus* shows obvious seasonal variation in feeding and body weight. Here, 24 fecal samples (8 per season) were collected from wild badgers at Culai Mountain (Shandong) in spring, summer and autumn. Untargeted metabolomics based on liquid chromatography–mass spectrometry (LC-MS) was combined with multivariate statistical models including principal component analysis (PCA), partial least-squares discriminant analysis (PLS-DA), and KEGG pathway functional annotation to systematically investigate gut metabolite composition, seasonal variations, and core regulatory pathways across the three seasons. Lipids and lipid-like molecules dominated the fecal metabolome. Summer metabolic profiles were clearly separated from the more similar spring and autumn profiles. Spring samples were enriched in putative tissue repair-related metabolites; summer samples accumulated fatty acyls and plant secondary metabolites; autumn samples were marked by antioxidant and energy-storage markers. Core pathways including α-linolenic acid, linoleic acid, riboflavin and tryptophan metabolism mediated seasonal physiological adjustment. This study reveals gut metabolic remodeling for seasonal adaptation and provides molecular evidence for mustelid conservation research. As no independent functional validation was performed, the proposed functional interpretations of the differential metabolites remain tentative hypotheses to be tested through targeted metabolomics and multi-omics integration.

## Simple Summary

Seasonal changes in temperature and food resources strongly affect the physiology of wild hibernating mammals. The Asian badger (*Meles leucurus*) accumulates fat in autumn for winter hibernation; however, how gut metabolites change across seasons remains poorly understood. In this work, we collected fecal samples of wild Asian badgers in spring, summer and autumn and performed untargeted metabolomics analysis. We found that summer gut metabolic profiles were distinctly different from spring and autumn. Spring samples were enriched with metabolites putatively linked to tissue repair after hibernation; summer samples contained abundant plant-derived substances reflecting diverse summer foods; autumn samples featured antioxidant-related metabolites putatively supporting pre-hibernation energy storage. Important metabolic pathways including linoleic acid metabolism and tryptophan metabolism mediated these seasonal physiological adjustments. This study improves our understanding of seasonal adaptation in hibernating mustelids and provides baseline data for wild badger conservation.

## 1. Introduction

Seasonal transitions bring periodic changes in temperature and food resources, which continuously disrupt the nutritional balance and physiological homeostasis of wild mammals. Gut metabolites are jointly produced by the host’s intestinal epithelial cells, symbiotic microbiota, and dietary substrates. They include diverse bioactive compounds such as short-chain fatty acids, bile acid derivatives, amino acid derivatives, and plant secondary metabolites [1,2]. These metabolites mediate multiple physiological processes, including intestinal homeostasis [3], immunomodulation, and energy metabolism, and act as key intermediates linking the external environment, microbial communities, and host phenotype [4].

*Meles leucurus* (Carnivora: Mustelidae) is a small- to medium-sized burrowing hibernating mammal [5]. Adults weigh 3.5–9 kg and exhibit pronounced seasonal weight gain together with winter hibernation [6,7,8]. This species performs important ecological functions in forest ecosystems. Its burrowing activity improves soil aeration, and abandoned burrows provide shelters for diverse small mammals [9]. Existing field surveys have confirmed that *Meles leucurus* is an omnivorous animal, with significant seasonal and geographic differences in food composition [10]; insects, small vertebrates, and wild plant roots, rhizomes, and fruits serve as the main food sources during different periods [5]. Studies have demonstrated that *Meles leucurus* shows marked seasonal nutritional adaptation: it accumulates large fat reserves in autumn to sustain hibernation at reduced metabolic rates, and its gut microbiota shifts with diet and season to enhance energy use efficiency [11].

Studies on intestinal microbiota further indicate that Firmicutes and Proteobacteria are year-round dominant taxa in the badger gut, with community structure undergoing marked remodeling according to season and food resources [12]. Nevertheless, current studies are limited to microbial community composition [13]. The patterns of small-molecule metabolic responses mediated by gut microbiota, as well as the metabolic regulatory pathways governing seasonal physiology, remain poorly understood, which hinders a full mechanistic understanding of its hibernation adaptation strategy.

In recent years, multi-omics technology has been widely applied in research on environmental adaptation of wild mammals. Studies targeting species such as *Przewalskium albirostris* [14] and *Spermophilus dauricus* [15,16] have confirmed that winter low temperatures and food scarcity drive directional remodeling of gut microbiota and metabolic pathways. Studies on migratory birds report convergent shifts in gut microbiota during energy reserve phases, and these shifts are directly correlated with individual body mass and fat deposition traits [17]. Research on the molecular mechanisms of host–microbe interactions further demonstrates that gut microbiota-derived metabolites modulate intestinal epithelial repair [11] and systemic energy metabolism [18] through HDAC3 and the bile acid FXR/TGR5 signaling axis, offering solid theoretical support for the present work.

Current research on the seasonal adaptation of *Meles leucurus* lacks systematic analysis at the metabolomic level, making it impossible to clarify the dynamic differentiation patterns of gut metabolites across different seasons. Based on this, the present study collected fecal samples from wild badgers in spring, summer, and autumn, and conducted analysis relying on LCMS untargeted metabolomics technology, with three research objectives: ① to systematically characterize the overall composition and seasonal differentiation characteristics of gut metabolites in *Meles leucurus*; ② to screen for marker differential small metabolites in each season; and ③ to identify the core KEGG metabolic pathways regulating seasonal physiological adaptation. The results of this study can improve the gut metabolome data repository of mustelid hibernating animals and enrich the theory of environmental adaptation in wild mammals from the perspective of “host–microbiota–metabolite” interactions, providing a molecular basis for wild badger population conservation.

## 2. Materials and Methods

### 2.1. Animals and Sample Collection

Fresh fecal samples from wild *Meles leucurus* were collected at Shandong Culai Mountain from 2022 to 2023, with sampling conducted across three seasons—spring, summer, and autumn—yielding 8 samples per season for a total of 24 samples. During sampling, a minimum distance of 200 m was maintained between samples to avoid repeated sampling from the same individual. Because Asian badgers, like other Eurasian badgers, defecate predominantly at latrines that are visited and shared by all members of a social group [19,20,21], this distance criterion, which was adopted based on field experience and the movement ecology of the species—individual home ranges in the genus *Meles* typically span tens of hectares and may expand further during the mating season [22,23]—rather than on individual identification, cannot entirely exclude repeated sampling of the same individual or of different individuals sharing the same latrine [20]; we therefore acknowledge this as a limitation of the study design. The freshness of each fecal sample was judged in the field using standardized physical criteria (dark color, moist glossy surface, soft consistency, and absence of drying, mold growth, or invertebrate colonization). The sampling sites lie within the Culai Mountain Nature Reserve (approximately 36.03° N, 117.33° E) in Tai’an, Shandong Province, a temperate deciduous broad-leaved forest habitat. Fresh fecal samples were immediately placed into sterile cryopreservation tubes after collection, transported back to the laboratory in a cryogenic insulated box, and, after passing quality inspection, stored long-term in a −80 °C ultra-low-temperature freezer at Novogene Co., Ltd. (Beijing, China). No field sampling procedures involved the capture or injury of wild animals and caused no disturbance to the native habitat. The animal experiment protocol was approved by the Biomedical Ethics Committee of Qufu Normal University and complied with relevant domestic laws and regulations on animal protection. All sampling procedures followed the ARRIVE 2.0 guidelines.

### 2.2. Sample Pretreatment

Each frozen fecal sample (100 mg) was fully ground under liquid nitrogen and extracted with 500 μL of 80% methanol (sample-to-solvent ratio 1:5, *w*/*v*); the mixture was vortexed and kept on ice for 5 min. After centrifugation at 15,000× *g* for 20 min at 4 °C, the supernatant was diluted with LC-MS-grade water to a final methanol concentration of 53% and centrifuged again under the same conditions prior to injection. A pooled quality control (QC) sample was prepared by mixing equal volumes of all experimental samples, and QC aliquots were injected at intervals throughout the analytical sequence to monitor instrumental stability; all 24 samples were measured within a single analytical batch. Analytical precision was evaluated using the QC injections: the median QC coefficient of variation (CV) of the annotated features was 10.7% in positive-ion mode and 9.5% in negative-ion mode, and 92.6% and 94.8% of the features, respectively, had a QC CV below 30%. The blank control consisted of 53% aqueous methanol and underwent identical pretreatment to remove interference from solvent impurities.

### 2.3. LC-MS Detection Conditions

The chromatographic system employed Thermo Vanquish ultra-high-performance liquid chromatography system (Thermo Fisher Scientific, Bremen, Germany), with a Hypersil Gold C18 column (100 × 2.1 mm, 1.9 μm; Thermo Fisher Scientific, Waltham, MA, USA), a column temperature of 40 °C, and a flow rate of 0.2 mL/min. Mobile phase B was methanol in both modes; mobile phase A was 0.1% formic acid in water in positive-ion mode and 5 mM ammonium acetate (pH 9.0) in negative-ion mode. The mass spectrometer was a Q Exactive HF/HFX high-resolution mass spectrometer (Thermo Fisher Scientific, Bremen, Germany), and metabolic mass spectrometry data were acquired in both positive and negative electrospray ionization (ESI) modes. The binary gradient elution program was as follows: 0–1.5 min, 2% B; 3 min, 85% B; 10 min, 100% B; 10.1–12 min, 2% B (re-equilibration), giving a total runtime of 12 min. The ESI source parameters were as follows: spray voltage 3.5 kV; sheath gas flow rate 35 psi; auxiliary gas flow rate 10 L/min; capillary (ion transfer tube) temperature 320 °C; S-lens RF level 60; auxiliary gas heater temperature 350 °C; full-scan range *m*/*z* 100–1500; MS/MS spectra were acquired in data-dependent acquisition mode.

### 2.4. Data Preprocessing and Statistical Analysis

Raw mass spectrometry data were imported into Compound Discoverer 3.3 for peak picking, retention time alignment, MS fragment matching, and preliminary metabolite annotation. Substances were matched based on the mzCloud and mzVault databases, and classification and pathway annotation were completed using the HMDB, LIPID MAPS, and KEGG databases. Metabolite abundances used in all statistical analyses were relative abundances (normalized peak areas) rather than absolute quantitative values. Statistical analyses and visualizations were performed with the metaX package in R (version 3.4.3) using the ggplot2 (https://cran.r-project.org/package=ggplot2, accessed on 7 October 2026), pheatmap (https://cran.r-project.org/package=pheatmap, accessed on 7 October 2026), and corrplot (https://cran.r-project.org/package=corrplot, accessed on 7 October 2026) packages, with additional data processing in Python (versions 2.7.6 and 3.5.0); KEGG enrichment analysis was performed in R (version 4.0.3). The confidence level of metabolite annotations was evaluated according to the Metabolomics Standards Initiative (MSI) criteria [24]; because no authentic chemical standards were analyzed, no metabolite reached MSI Level 1; features supported by MS/MS spectral matches (mzCloud or mzVault full match) were classified as MSI Level 2 (491 of 1173 features, 41.9%, in positive-ion mode; 445 of 815, 54.6%, in negative-ion mode), whereas features matched by accurate mass alone were regarded as Level 3–4 (putatively characterized compound classes or unknown features). Multivariate statistics employed PCA and PLS-DA models, with 7-fold cross-validation and a 200-permutation test to verify model stability. Differential metabolite screening criteria were VIP > 1, *p* < 0.05, and FC > 1.2 or FC < 0.833 (the criteria used for all figures and counts reported in this study). Benjamini–Hochberg false discovery rate (FDR) correction was additionally applied to the *p* values of all features within each comparison and ion mode (*n* = 8 per season), and the resulting q values are reported for every differential feature in the results below.

## 3. Results

### 3.1. Data Quality Control and Multivariate Statistical Analysis

Pearson correlation coefficients were computed using metabolite relative abundance values (normalized peak intensities rather than absolute concentrations) from all quality control (QC) samples. QC sample correlation coefficients approached 1 in both positive and negative-ion modes (Supplementary Figure S1), indicating satisfactory instrumental stability for LCMS detection and high data reliability.

**Figure 1:**
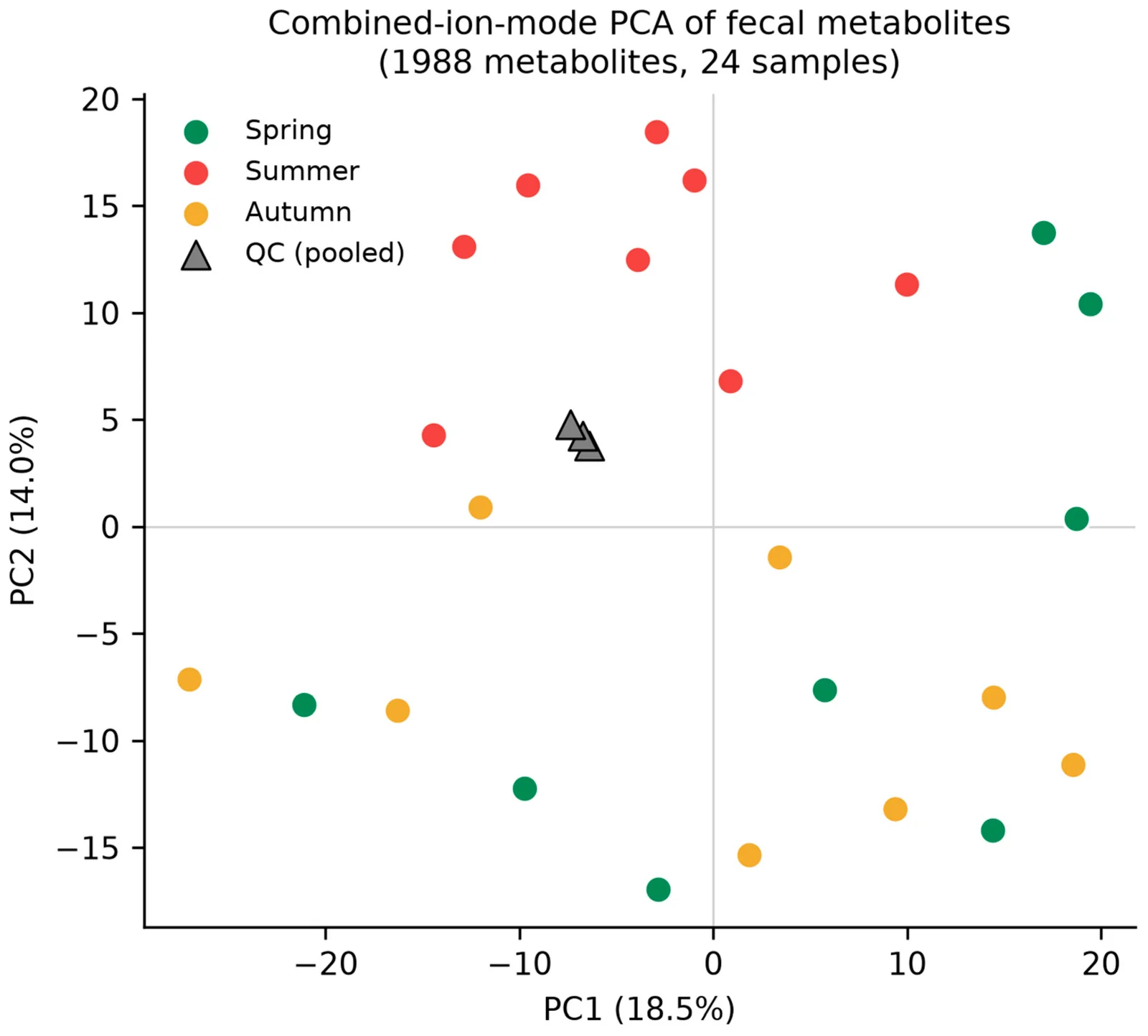
PCA score plot of fecal metabolites in combined ion modes.

To intuitively present the overall metabolome differences among the three groups of samples, principal component analysis (PCA) was conducted. In positive-ion mode, summer samples clustered independently, while spring and autumn samples showed a higher degree of spatial overlap. In negative-ion mode, the discrimination among samples decreased slightly, but a consistent grouping trend was still maintained (Supplementary Figure S2). When the two ionization modes were combined (each metabolite represented by its most abundant peak across modes), the PCA separated the summer group from the spring and autumn groups along the first two principal components, which explained 18.5% and 14.0% of the total variance, respectively, while the pooled quality control samples clustered tightly near the origin, confirming acceptable analytical stability (Figure 1). These findings demonstrate that season is the core factor driving changes in the composition of gut metabolites in badgers, with higher similarity in metabolic profiles between spring and autumn, and specific metabolic characteristics in summer.

A partial least-squares discriminant analysis (PLSDA) model was further applied to conduct discrimination between groups. Effective separation was achieved in all three pairwise comparisons: spring vs. autumn, spring vs. summer, and summer vs. autumn. Two-hundred permutation tests revealed that the original R^2^ and Q^2^ values for each model were substantially higher than those for the permuted datasets, confirming no overfitting and reliable explanatory and predictive performance (Supplementary Figures S3 and S4).

### 3.2. Overall Compositional Characteristics of Gut Metabolites

#### 3.2.1. HMDB Annotation of Metabolite Categories

**Figure 2:**
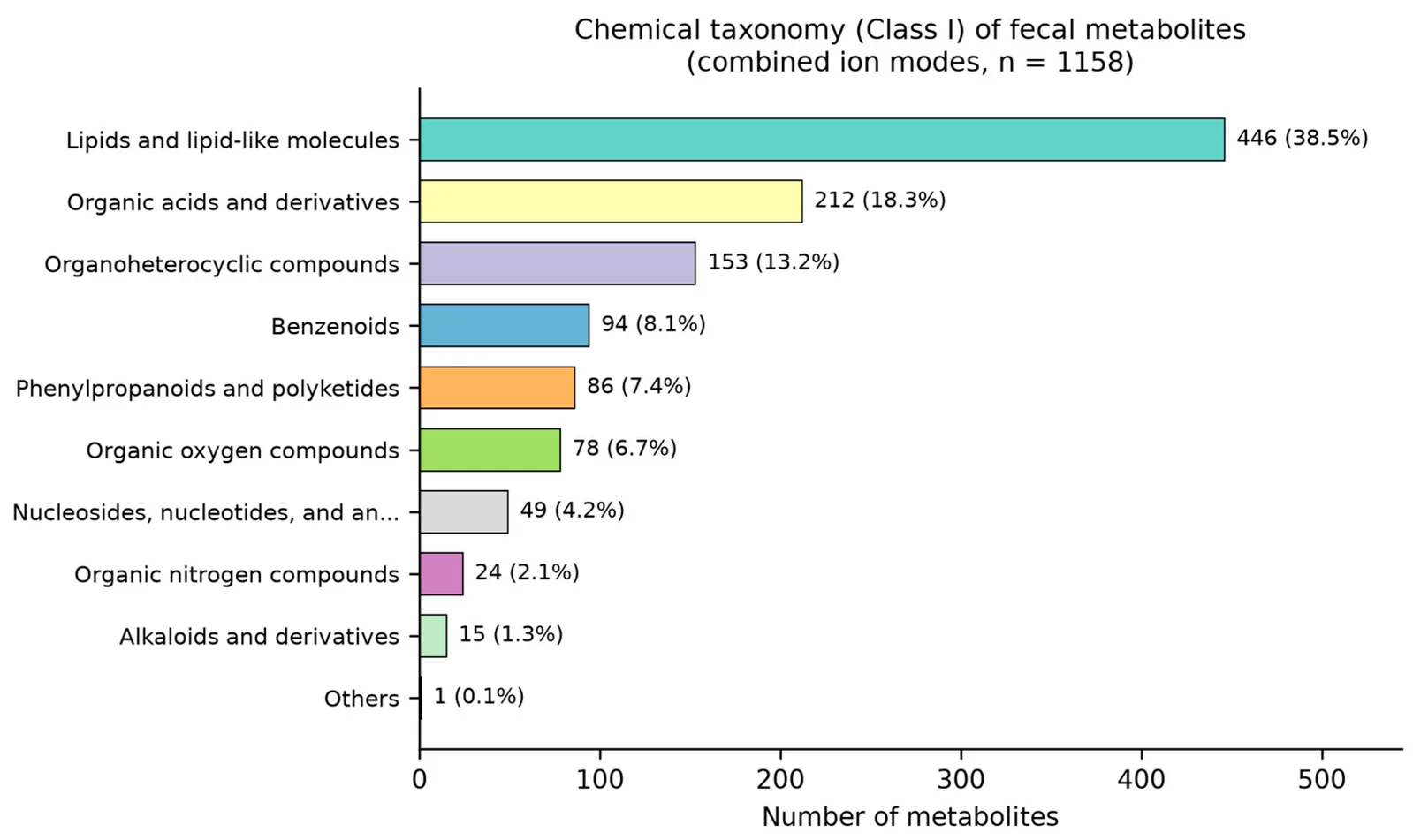
Chemical taxonomy (Class I) classification of fecal metabolites in combined ion modes.

In positive-ion mode, 1173 metabolites were annotated; 815 were annotated in negative-ion mode. Chemical taxonomy classification (Class I) showed that lipids and lipid-like molecules constituted the most abundant class under both detection modes (33.93% of taxonomically assigned features in positive-ion mode, 43.67% in negative-ion mode), followed by organic acids and derivatives (17.62% and 19.08%, respectively). Consistently, HMDB superclass annotation of the 473 positive-ion and 375 negative-ion features with HMDB entries likewise ranked lipids and lipid-like molecules first (29.39% and 32.27%), followed by organic acids and derivatives (19.03% and 22.93%). This compositional feature matches the physiological characteristic of substantial fat storage in badgers before hibernation. When the two ionization modes were merged, 1158 metabolites were assigned to a chemical class, and lipids and lipid-like molecules again ranked first (446 metabolites, 38.5%), followed by organic acids and derivatives (212, 18.3%) and organo-heterocyclic compounds (153, 13.2%) (Figure 2).

#### 3.2.2. Detailed LIPID MAPS Annotation of Lipids

**Figure 3:**
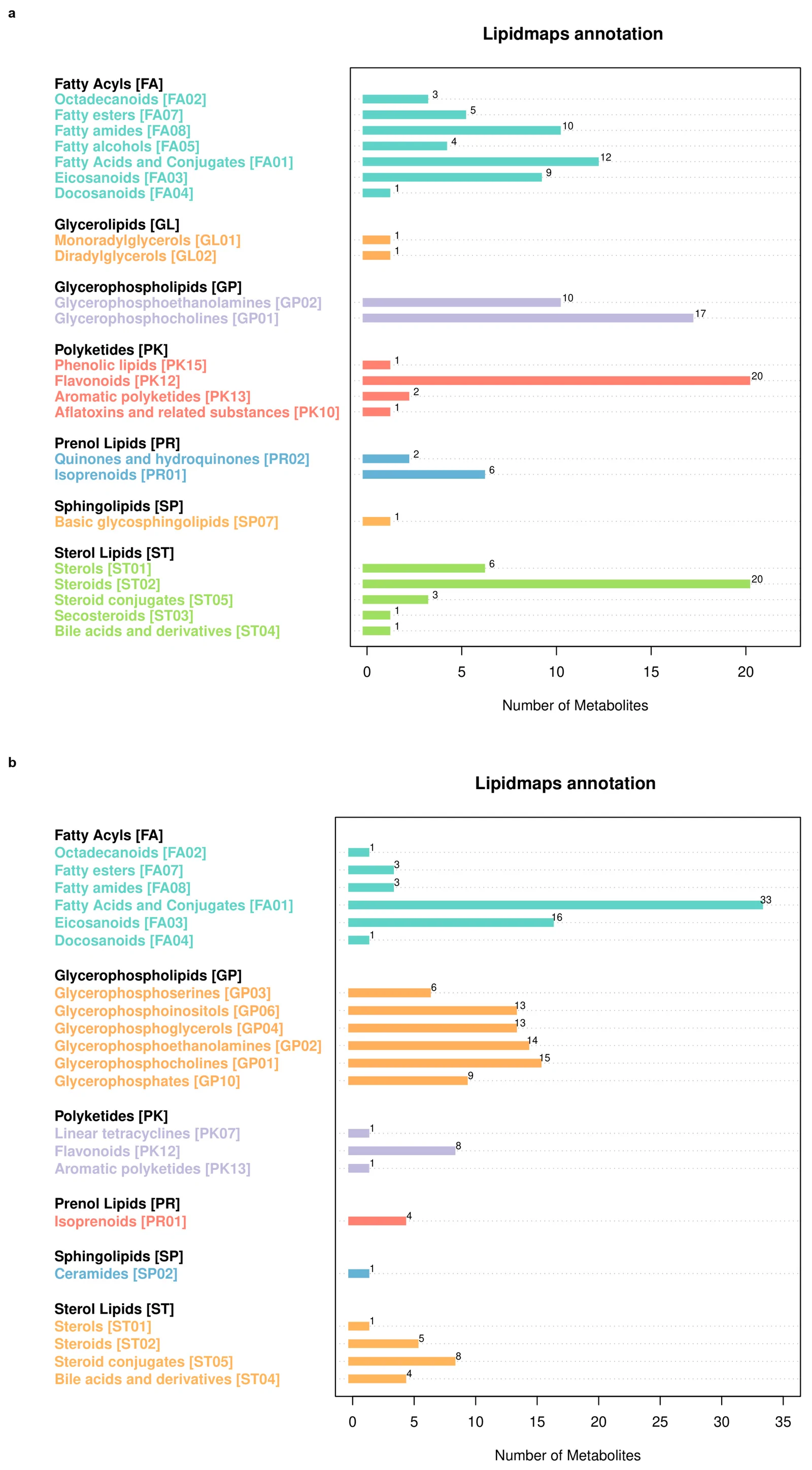
Lipid classification by LIPID MAPS for badger feces: (**a**) positive-ion mode; (**b**) negative-ion mode.

Lipid subclasses were further annotated in detail based on the LIPID MAPS database. Steroids and glycerophosphocholines were detected in the greatest numbers in positive-ion mode, while negative-ion mode showed higher detection sensitivity for fatty acids and conjugates, with a total of 33 fatty acid-related substances annotated (Figure 3).

#### 3.2.3. Global KEGG Pathway Annotation

**Figure 4:**
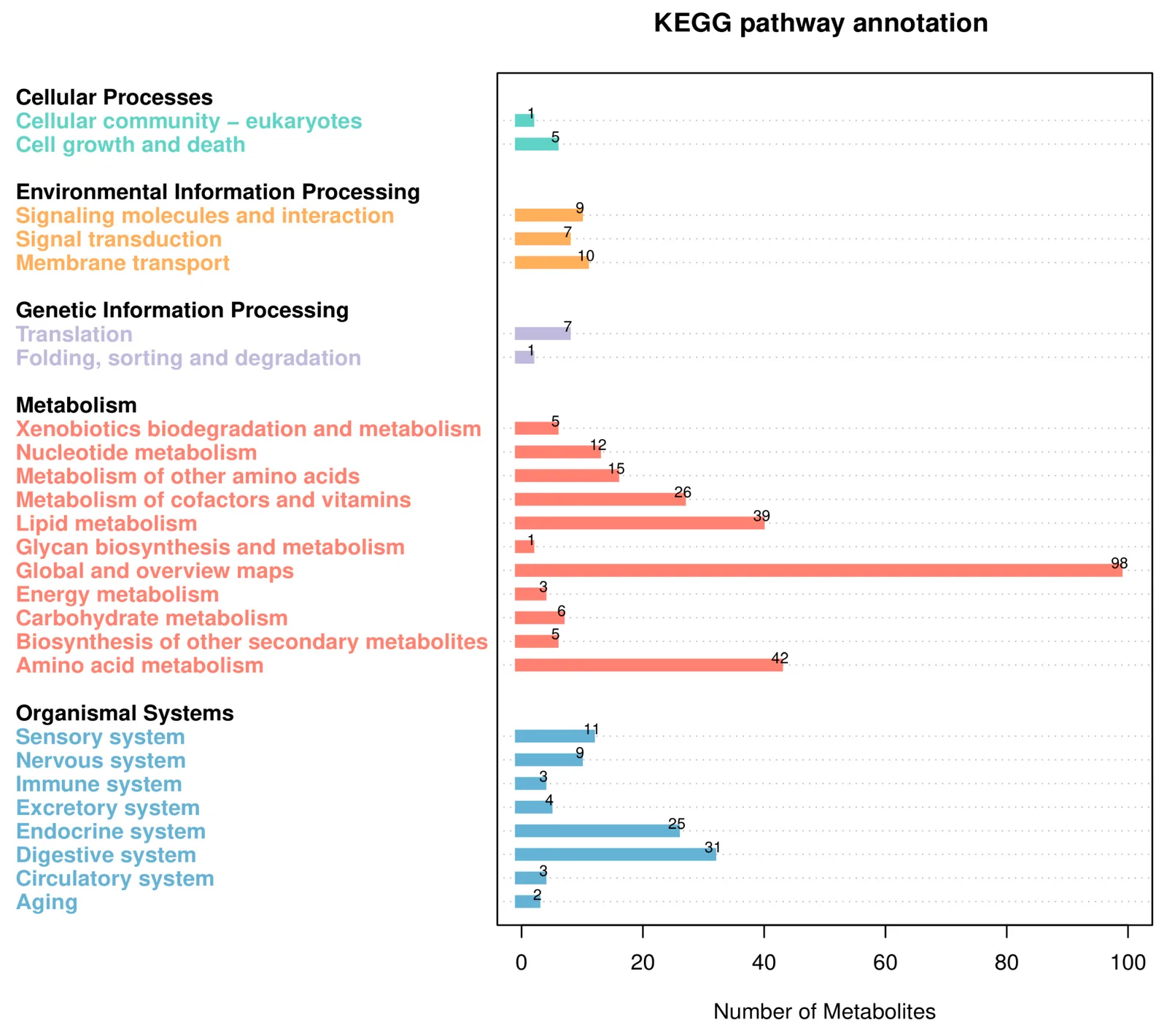
KEGG pathway annotation of metabolites in positive-ion mode.

**Figure 5:**
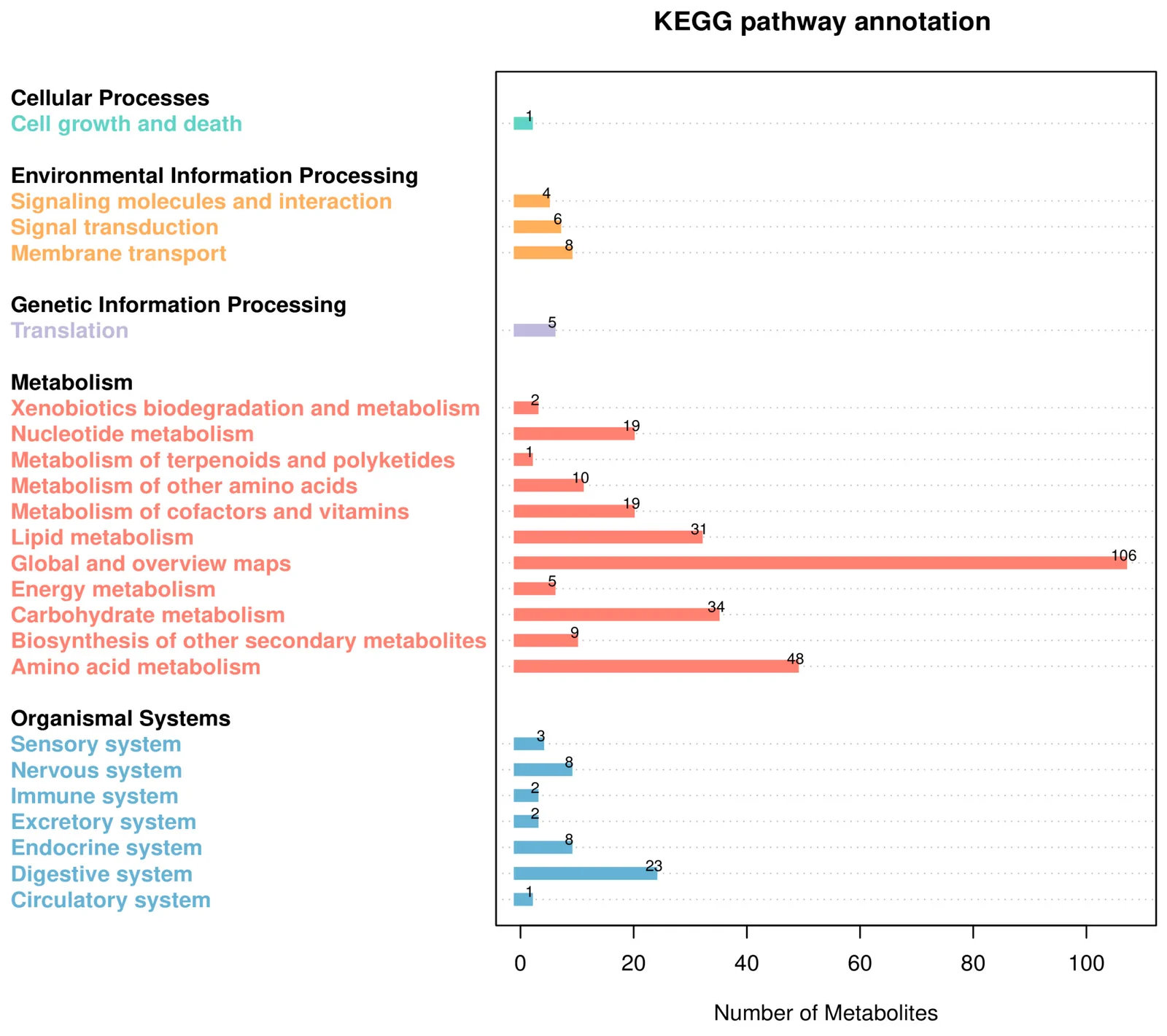
KEGG pathway annotation of metabolites in negative-ion mode.

The results of KEGG level 1 pathway annotation showed that metabolism-related pathways were annotated with the largest number of metabolites, among which global metabolic overview, amino acid metabolism, and lipid metabolism accounted for the highest proportions. Pathways related to the digestive system were also markedly enriched, suggesting sustained high nutrient metabolic activity in the badger gut across seasons (Figure 4 and Figure 5).

### 3.3. Analysis of Seasonal Differential Metabolites

#### 3.3.1. Distribution of Number of Differential Metabolites

**Figure 6:**
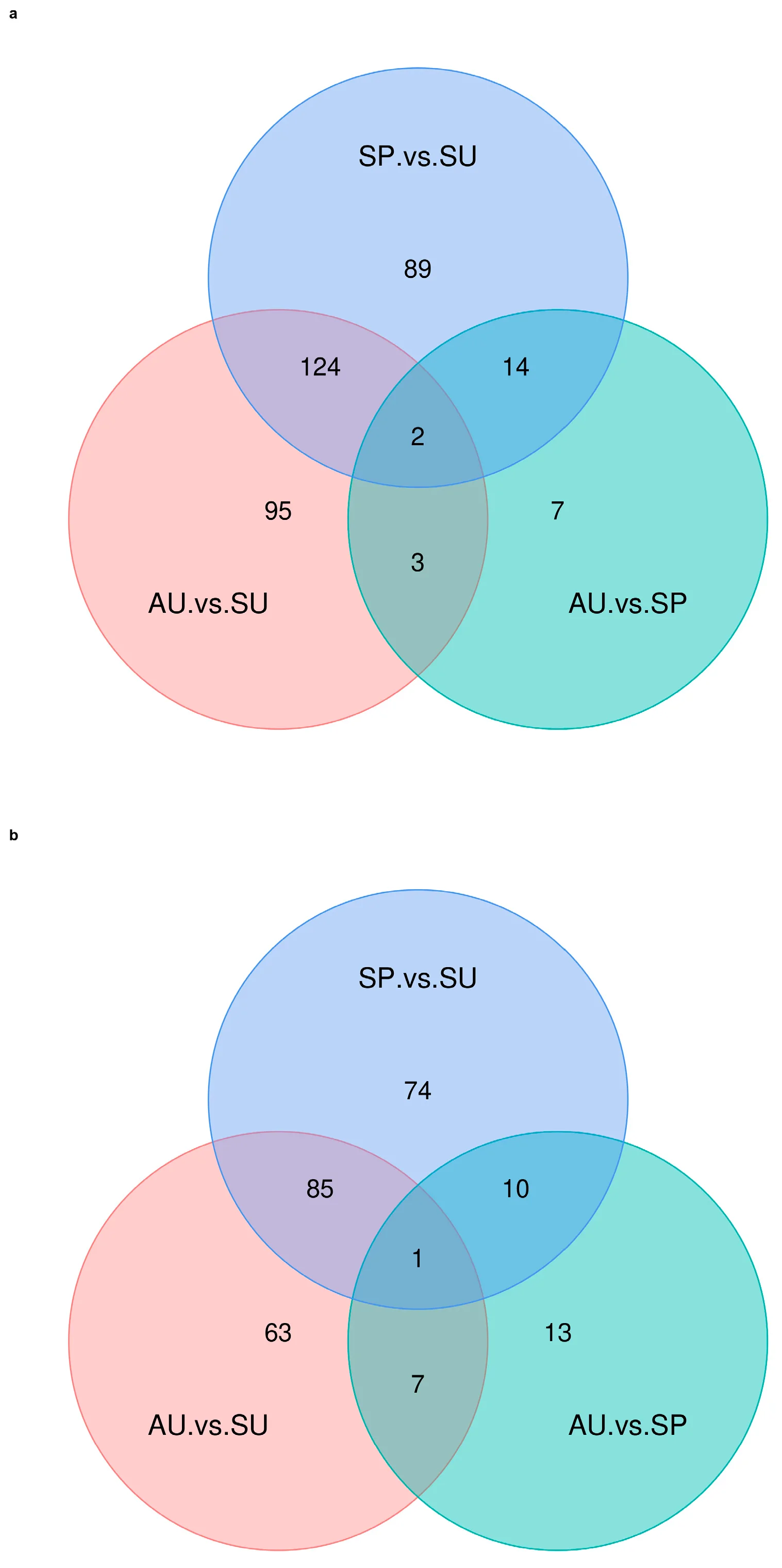
Venn plots of seasonal differential metabolites: (**a**) positive-ion mode; (**b**) negative-ion mode.

Differential metabolites were screened with criteria VIP > 1, *p* < 0.05, and FC > 1.2 or FC < 0.833. Positive-ion mode yielded 229 differential metabolites in the spring-versus-summer comparison, 224 in the autumn-versus-summer comparison and 26 in the autumn-versus-spring comparison (union: 334); the corresponding numbers in negative-ion mode were 170, 156 and 31 (union: 253). Benjamini–Hochberg correction showed that 101 of the differential metabolites of the spring-versus-summer comparison (59 in positive-ion and 42 in negative-ion mode) and 67 of those of the autumn-versus-summer comparison (24 and 43, respectively) retained q < 0.05, whereas none of the 57 differential metabolites of the autumn-versus-spring comparison did, indicating that the autumn–spring differences are suggestive rather than definitive. Venn diagram results showed that the number of unique differential metabolites in the spring vs. summer and autumn vs. summer groups was far greater than that in the spring vs. autumn group, further confirming that the summer metabolome differed significantly from the spring and autumn groups, while the metabolic characteristics of spring and autumn were more similar (Figure 6).

#### 3.3.2. Volcano Plot Analysis of Differential Metabolite Abundance

**Figure 7:**
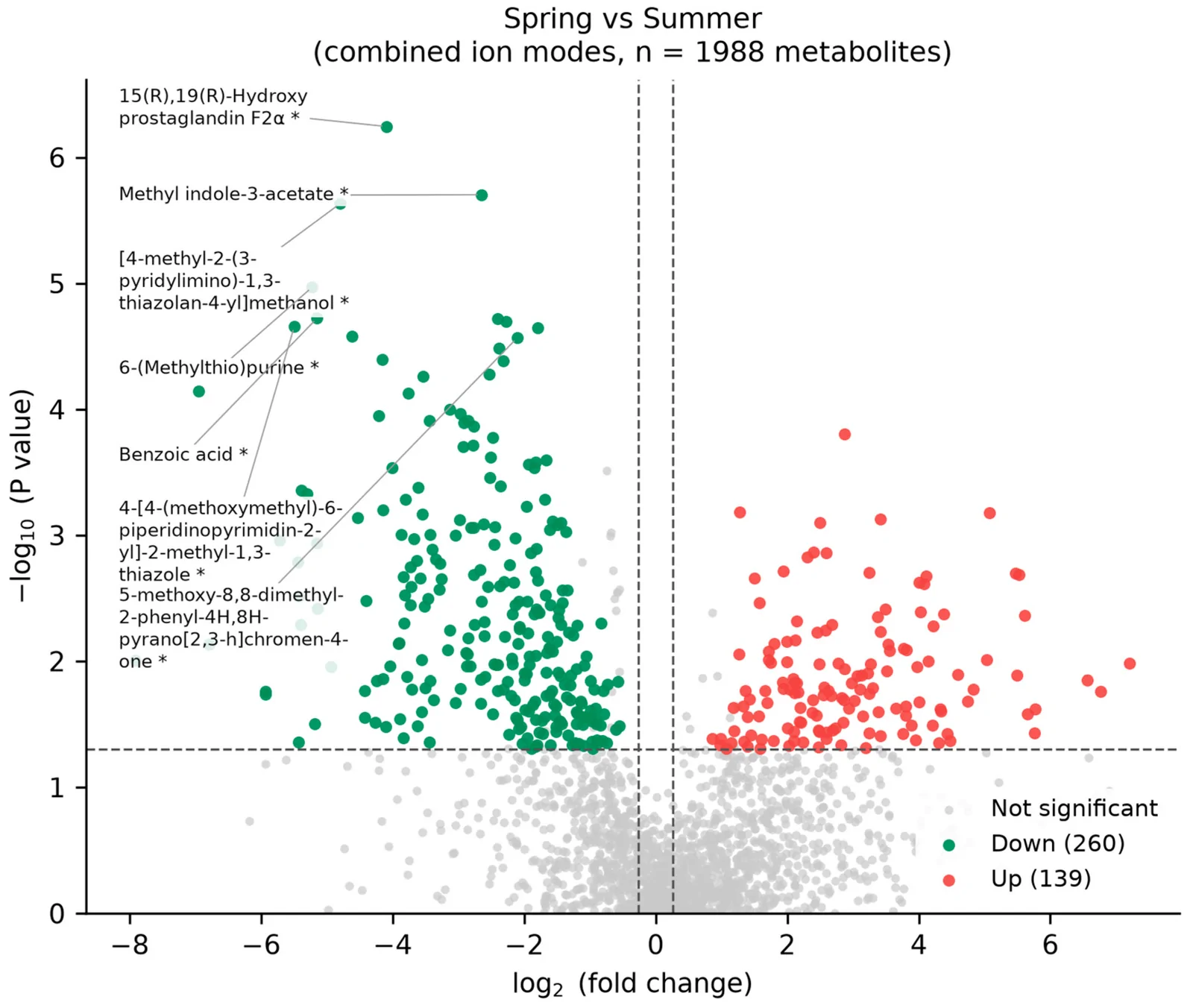
Volcano plot of differential metabolites between spring and summer groups in combined ion modes. Asterisks (*) mark metabolites with FDR-adjusted q < 0.05.

**Figure 8:**
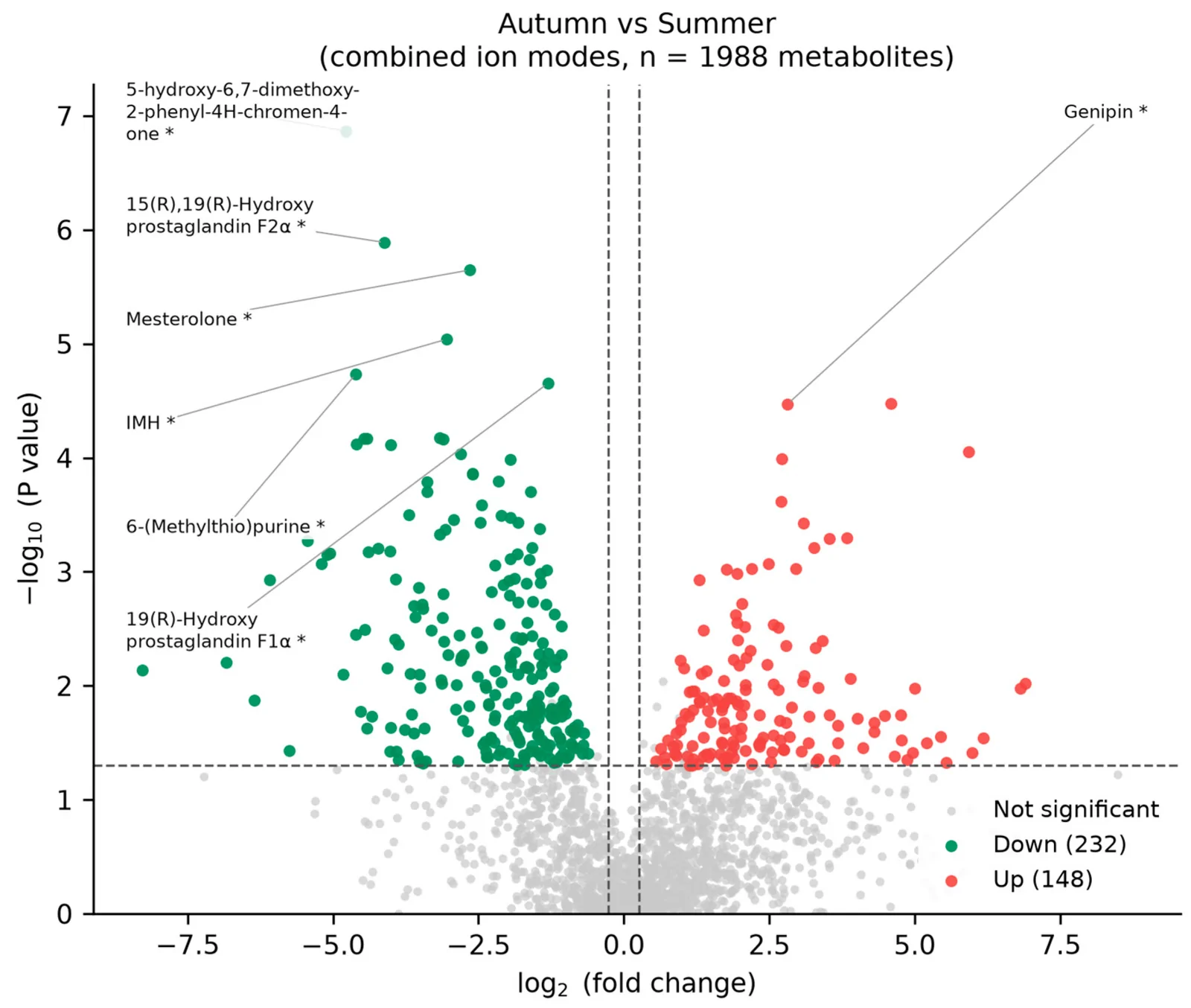
Volcano plot of differential metabolites between autumn and summer groups in combined ion modes. Asterisks (*) mark metabolites with FDR-adjusted q < 0.05.

**Figure 9:**
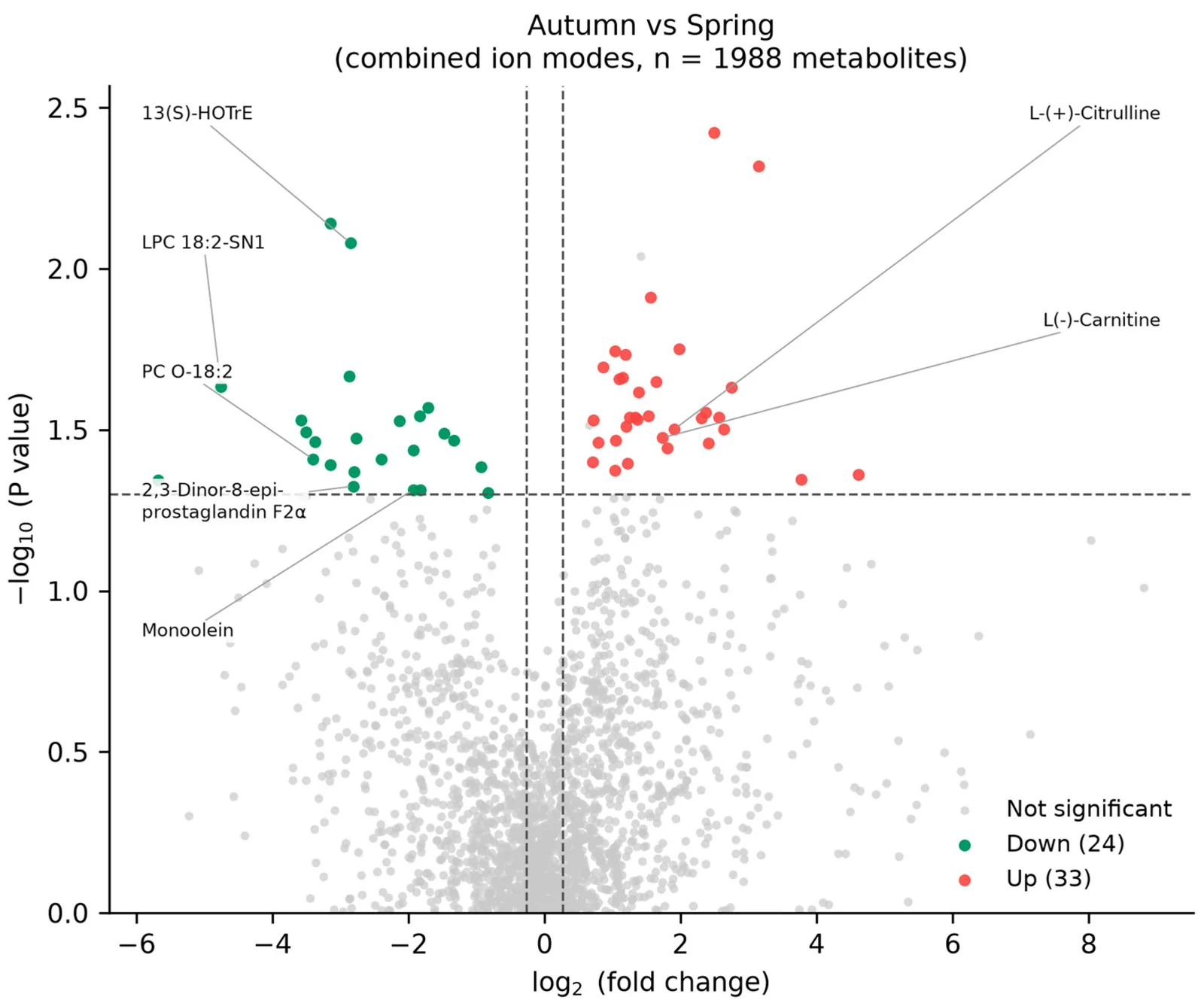
Volcano plot of differential metabolites between autumn and spring groups in combined ion modes.

Volcano plot analyses indicated that, relative to summer samples, spring and autumn groups yielded substantially more down-regulated than up-regulated differential metabolites in positive-ion mode (183 versus 46 in the spring-versus-summer comparison and 163 versus 61 in the autumn-versus-summer comparison), whereas negative-ion mode showed the opposite trend, with more up-regulated than down-regulated metabolites (93 versus 77 and 87 versus 69, respectively). When the two ionization modes were combined, the spring-versus-summer comparison yielded 139 up-regulated and 260 down-regulated differential metabolites, of which 101 were significant at FDR q < 0.05 (Figure 7), and the autumn-versus-summer comparison yielded 148 up-regulated and 232 down-regulated differential metabolites, of which 67 were significant at q < 0.05 (Figure 8); the autumn-versus-spring comparison yielded only 33 up-regulated and 24 down-regulated differential metabolites, none of which survived FDR correction (Figure 9), again indicating that the spring and autumn metabolic profiles were the most similar.

#### 3.3.3. Seasonally Characteristic Marker Metabolites

**Figure 10:**
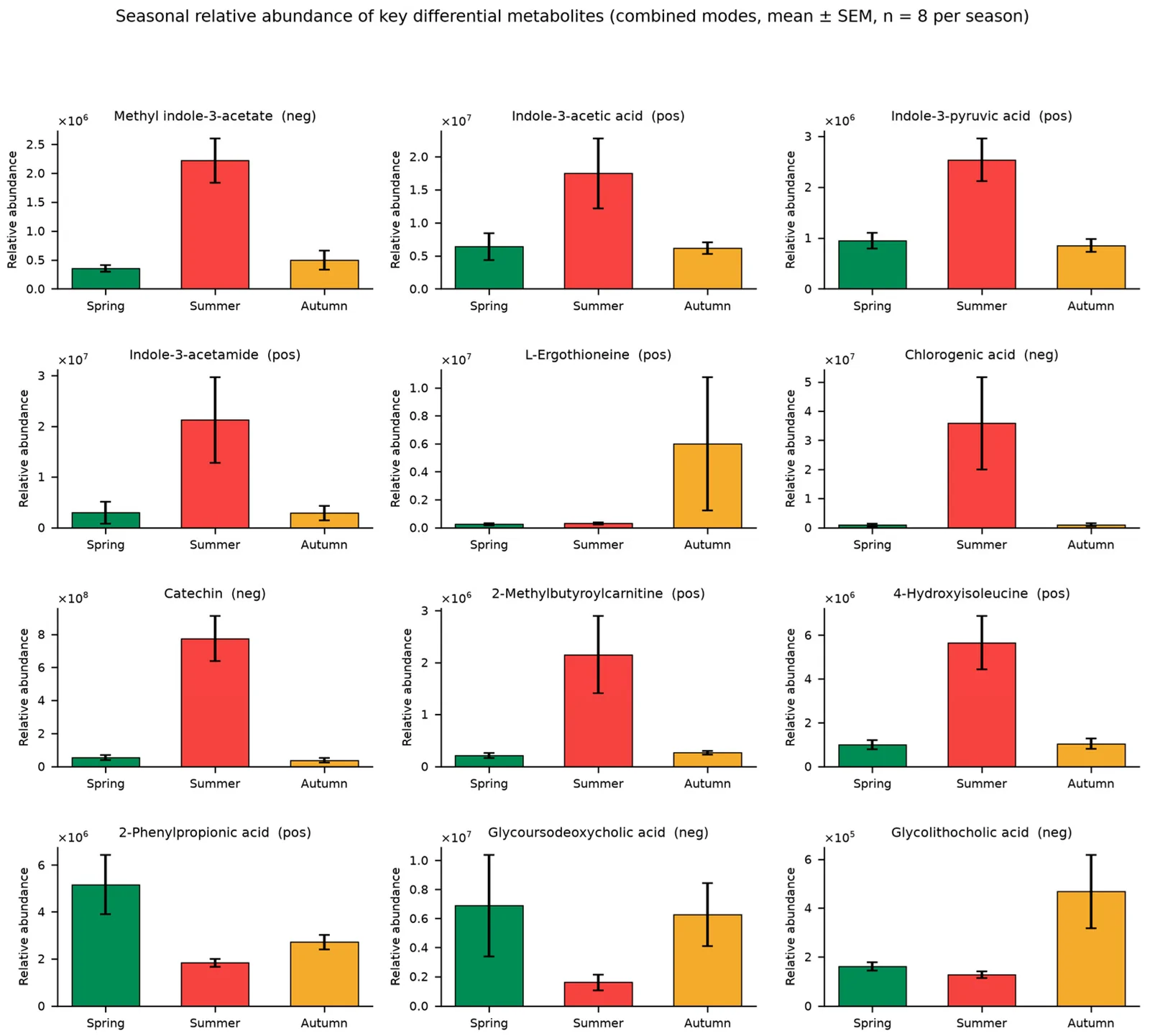
Seasonal relative abundance of key differential metabolites (combined ion modes).

Stick plot and hierarchical clustering heatmap results showed clear distinctions in characteristic metabolites among the seasons. High-abundance substances in spring samples were predominantly glycerophospholipids and phenolic plant metabolites such as lysophosphatidylcholine species (LPC 18:2, LPC 16:0 and LPC 20:4), 2-phenylpropionic acid, and ellagic acid, which were also elevated in autumn relative to summer, mainly involved in cell membrane synthesis and tissue damage repair. Summer samples were enriched in fatty acyls and plant secondary metabolites such as D-2-aminoadipic acid, gramine, and 6-Sialyllactose, directly associated with diverse summer feeding sources. Autumn samples were specifically enriched in antioxidant and lipid transport small molecules such as *L*-ergothioneine, vitamin B2, and *L*-citrulline, suited to energy storage and antioxidation needs before hibernation. The seasonal patterns of these key differential metabolites are shown as mean ± SEM bar plots in Figure 10.

The hierarchical clustering heatmap showed that spring and autumn samples clustered into the same branch, while summer samples clustered separately, corroborating the conclusions from PCA and the Venn diagram (Supplementary Figures S5–S11).

#### 3.3.4. KEGG Differential Pathway Enrichment Analysis

The results of the KEGG pathway enrichment bubble chart between groups showed that in the autumn-versus-spring comparison—the comparison with the fewest differential metabolites and no FDR-significant features—alpha-linolenic acid metabolism (positive-ion mode, *p* = 0.013), linoleic acid metabolism (negative-ion mode, *p* = 0.046), and riboflavin metabolism (negative-ion mode, *p* = 0.046) were the enriched pathways, each represented by a single metabolite; so, these results should be regarded as indicative rather than conclusive. In the comparisons involving summer, tryptophan metabolism was significantly enriched in positive-ion mode (spring versus summer, *p* = 4.2 × 10^−4^, six metabolites including L-kynurenine, indole-3-acetic acid, and indole-3-acetamide; autumn versus summer, *p* = 0.019, four metabolites), accompanied by differentiation in amino sugar and nucleotide sugar metabolism and vitamin B6 metabolism (spring versus summer) and in glyoxylate and dicarboxylate metabolism, C5-branched dibasic acid metabolism, and propanoate metabolism (autumn versus summer) (Supplementary Figures S12–S14).

#### 3.3.5. Screening of Known Microbiota-Derived Metabolite Classes

Because the fecal metabolome also integrates gut microbial activity, the annotated feature table was additionally screened for several well-characterized classes of microbiota-derived metabolites. Free short-chain fatty acids (acetate, propionate and butyrate) were not recovered by the present reversed-phase LC-MS platform and could therefore not be evaluated. Bile acids and their conjugates were represented by 14 annotated features in negative-ion mode, together with 10 further bile acid-related features in positive-ion mode; the negative-ion features comprised free cholic, chenodeoxycholic, deoxycholic, lithocholic and β-muricholic acids together with their glycine-, taurine- and sulfate-conjugated forms. Only two features differed significantly between seasons (glycoursodeoxycholic acid, higher in spring than in summer, FC = 4.29, *p* = 0.043; glycolithocholic acid, higher in autumn than in summer, FC = 3.68, *p* = 0.031), and neither passed the FDR threshold (q = 0.21 and 0.22). Branched-chain amino acid catabolites were more clearly affected: in positive-ion mode, 2-methylbutyroylcarnitine and 4-hydroxyisoleucine were both markedly lower in spring and autumn than in summer (q = 0.040 and 0.019 for spring versus summer). Among aromatic microbial metabolites, 2-phenylpropionic acid (positive-ion mode) was significantly higher in spring than in summer (FC = 2.82, *p* = 0.002, q = 0.046), whereas the polyamine conjugate N-p-coumaroyl spermidine was about 17-fold higher in summer (FC = 0.06, *p* = 0.011 for spring versus summer).

## 4. Discussion

This study confirms that lipids and lipid-like molecules constitute the dominant metabolic category in the intestinal tract of badgers, a pattern consistent with that observed in hibernating or high-altitude wild mammals such as *Przewalskium albirostris* and *Spermophilus dauricus*. This represents an adaptive strategy formed through convergent evolution in hibernating animals: substantial intake of lipid substrates in autumn, followed by metabolic transformation via gut microbiota, results in fat storage that supports prolonged dormancy during winter. A large number of organic acids and plant secondary metabolites were detected in the samples, directly substantiating the broad omnivorous feeding characteristics of the badger [13,25], consistent with findings from studies on the feeding habits of European badgers. KEGG global annotation results showed sustained high activity in amino acid and lipid metabolism pathways, reflecting a relatively high intensity of energy turnover in this species throughout the year, matching the energy expenditure demands of burrowing activity and seasonal reproduction.

The combined results of all multivariate statistical analyses jointly demonstrate that spring and autumn metabolome profiles exhibit higher similarity, whereas summer gut metabolic characteristics show significant specificity. This pattern of differentiation is highly consistent with the dynamics of wild seasonal food resources. In summer, insect, berry, and amphibian food resources are abundant, and dietary substrate diversity is at its highest, inducing the generation of large amounts of plant secondary metabolites and tryptophan-derived metabolites. In spring and autumn, food types tend to become more uniform, and the physiological focus of individuals shifts toward fat storage [26]. This “summer-specific, spring–autumn convergent” pattern of metabolic differentiation contrasts with the phenomenon of convergent gut microbiota during the energy reserve phase in migratory birds [17], reflecting divergent evolutionary strategies of energy regulation between hibernating mammals and migratory birds.

Spring samples were enriched in glycerophospholipids, predominantly lysophosphatidylcholine species such as LPC 18:2, LPC 16:0 and LPC 20:4. Phospholipids are core building blocks of cell membranes. Following hibernation, the intestine and muscle tissues exhibit varying degrees of damage, and the body requires large quantities of phospholipids to complete cell proliferation and tissue repair [27]. This mechanism is analogous to the up-regulated intestinal phospholipid metabolism observed in postweaning lactating calves. Phospholipid signaling molecules derived from gut microbiota can act in concert with the HDAC3 pathway to promote intestinal epithelial repair [28], jointly accomplishing the body’s recovery after hibernation.

The high abundance of fatty acyl metabolites in summer suggests active lipid metabolism, which may be related to the high energy demand in summer and the physiological process of energy storage for autumn and winter [11]. The summer samples also showed pronounced enrichment of indole derivatives formed through microbial tryptophan transformation, including indole-3-acetic acid, indole-3-pyruvic acid, indole-3-acetamide and methyl indole-3-acetate, together with the plant-derived indole alkaloid gramine. Indole derivatives generated from tryptophan transformation by gut microbiota can regulate intestinal immune homeostasis [29]. Meanwhile, large amounts of plant antioxidants such as chlorogenic acid and catechin were detected in the samples, which can relieve the oxidative stress caused by high temperatures in summer and plant secondary toxins [30], thereby reducing damage to the organism.

Autumn-specific enrichment of the antioxidants *L*-ergothioneine and vitamin B2 [31] can relieve the oxidative stress induced by extensive lipolysis before hibernation. Enriched *L*-citrulline may participate in nitrogen metabolism [14,16], thereby enhancing fat storage efficiency. This adaptive strategy is highly consistent with the research findings on vitamin and antioxidant metabolite enrichment in *Przewalskium albirostris* during winter [14], demonstrating that enhanced antioxidant capacity is a common adaptive mechanism among hibernating animals prior to overwintering.

The results of KEGG pathway enrichment analysis revealed the core metabolic pathways underlying the seasonal adaptation of badgers. The metabolic pathways of α-linolenic acid and linoleic acid, two essential fatty acids, differed between the spring and autumn groups, although this difference remained indicative rather than robust to FDR correction when the two groups were compared directly. These plant-derived fatty acids can regulate lipid homeostasis, supporting tissue repair in spring and fat storage in autumn [31,32]. Tryptophan metabolism was the signature differential pathway in summer, with indole signaling molecules generated through microbial transformation linking dietary structure, gut microbiota, and host immunity [16,29]. Riboflavin, as a key cofactor for oxidoreductases, showed pathway enrichment in the autumn-versus-spring comparison, which may enhance energy metabolism efficiency and antioxidant defense capacity [33].

It should be noted that the riboflavin feature detected in positive-ion mode was in fact more abundant in summer, and the feature annotated as ergothioneine in negative-ion mode was likewise higher in summer, whereas the features annotated as vitamin B2 and ergothioneine in positive-ion mode were higher in autumn; the vitamin-related pathway signals should therefore be interpreted with caution. Integrating the differential metabolites with pathway enrichment results, a seasonal metabolic regulation framework for *Meles leucurus* can be constructed. In summer, animals activate tryptophan metabolism to cope with dietary diversity and high-temperature stress. In autumn, lipid and vitamin metabolic pathways are activated to complete energy reserve accumulation for hibernation. In spring, the phospholipid metabolic pathway is up-regulated to achieve whole-body tissue repair after hibernation.

This study collected fecal samples only for spring, summer, and autumn; no samples were obtained during winter hibernation. Therefore, it cannot fully capture annual metabolic dynamics. Only untargeted metabolomics analysis was conducted, without combining metagenome or targeted metabolomics to verify the causal association between gut microbiota and key metabolites. Future studies could supplement winter hibernation fecal samples to fully elucidate the annual gut metabolic cycle of *Meles leucurus.* Multi-omics data from gut metagenome and targeted metabolomics should be integrated to identify core functional microbial communities regulating seasonal adaptation. Captive controlled feeding experiments should be set up to separate temperature and food variables and analyze their independent effects on gut metabolism. Moreover, the age and sex of the sampled individuals could not be determined from non-invasively collected feces, so potential age and sex effects on the metabolomic profiles could not be statistically controlled; because metabolomic profiles are highly sensitive to age and sex, the observed seasonal differences may partly reflect demographic differences among samples. Finally, because no independent functional validation (e.g., targeted assays, gut metagenomic sequencing, or controlled feeding experiments) was performed, the functional interpretations of differential metabolites proposed in this study should be regarded as cautious hypotheses rather than demonstrated causal mechanisms. In addition, no differential metabolite of the autumn-versus-spring comparison remained significant after Benjamini–Hochberg correction, so the spring–autumn pathway differences should be interpreted as indicative rather than conclusive.

## 5. Conclusions

This study characterized seasonal variation in gut fecal metabolites of wild *Meles leucurus* using LCMS-based untargeted metabolomics. Lipid-related metabolites dominated the fecal metabolome across seasons. Summer exhibited distinct metabolic signatures, whereas spring and autumn shared more similar metabolite profiles. Key differential metabolites and KEGG pathways putatively reflected seasonal physiological demands: tissue repair in spring, response to diverse dietary resources in summer, and antioxidant defense and energy accumulation for hibernation in autumn. Limitations include the lack of winter hibernation samples and the absence of multi-omics validation. Future work should integrate metagenomics and targeted metabolomics together with winter samples to further decode host–microbe metabolic crosstalk during seasonal adaptation of badgers.

## Data Availability

The raw metabolomics data and metabolite annotation files generated in this study have been deposited in the MetaboLights database (EMBL-EBI) under the accession number MTBLS15895 (https://www.ebi.ac.uk/metabolights/MTBLS15895, accessed on 7 October 2026). The record is currently set to private with a scheduled public release on 1 December 2027.

## Supplementary Materials

The following supporting information can be downloaded at https://www.mdpi.com/article/10.3390/ani16203182/s1: Figure S1: Pearson correlation coefficient diagram of QC samples (a. positive-ion mode; b. negative-ion mode); Figure S2: PCA analysis diagram of metabolites (a. positive-ion mode; b. negative-ion mode); Figure S3: PLSDA analysis results among different groups (positive-ion mode on the left, negative-ion mode on the right); Figure S4: 200-permutation test results of PLSDA models among different groups (positive-ion mode on the left, negative-ion mode on the right); Figure S5: Matchstick diagram of differential metabolites between SP and SU groups (positive-ion mode); Figure S6: Matchstick diagram of differential metabolites between AU and SU groups (positive-ion mode); Figure S7: Matchstick diagram of differential metabolites between AU and SP groups (positive-ion mode); Figure S8: Matchstick diagram of differential metabolites between SP and SU groups (negative-ion mode); Figure S9: Matchstick diagram of differential metabolites between AU and SU groups (negative-ion mode); Figure S10: Matchstick diagram of differential metabolites between AU and SP groups (negative-ion mode); Figure S11: Clustering heatmap of total differential metabolites (a. positive-ion mode; b. negative-ion mode); Figure S12: Enrichment results of KEGG pathway between groups AU and SP (a. positive ion mode; b. negative ion mode); Figure S13: Enrichment results of KEGG pathway between groups SP and SU (a. positive ion mode; b. negative ion mode); Figure S14: Enrichment results of KEGG pathway between groups AU and SU (a. positive ion mode; b. negative ion mode).

## Abbreviations

The following abbreviations are used in this manuscript:

LCMS	Liquid Chromatography–Mass Spectrometry
PCA	Principal Component Analysis
PLSDA	Partial Least-Squares Discriminant Analysis
KEGG	Kyoto Encyclopedia of Genes and Genomes
QC	Quality Control
CV	Coefficient of Variation
ESI	Electrospray Ionization
HMDB	Human Metabolome Database
LIPID MAPS	LIPID Metabolites and Pathways Strategy
MSI	Metabolomics Standards Initiative
VIP	Variable Importance in Projection
FC	Fold Change
FDR	False Discovery Rate
