# Supplementary material for: Comparative Analysis of Gut Metabolites in Meles leucurus Across Different Seasons
Source: Animals (Basel). 2026 Oct 10;16(20):3182. doi: 10.3390/ani16203182 (PMC13700622; doi:10.3390/ani16203182)
Supplement: Supplementary file 1 [file animals-16-03182-s001.zip › animals-4556482-supplementary.pdf]

Supplementary Information

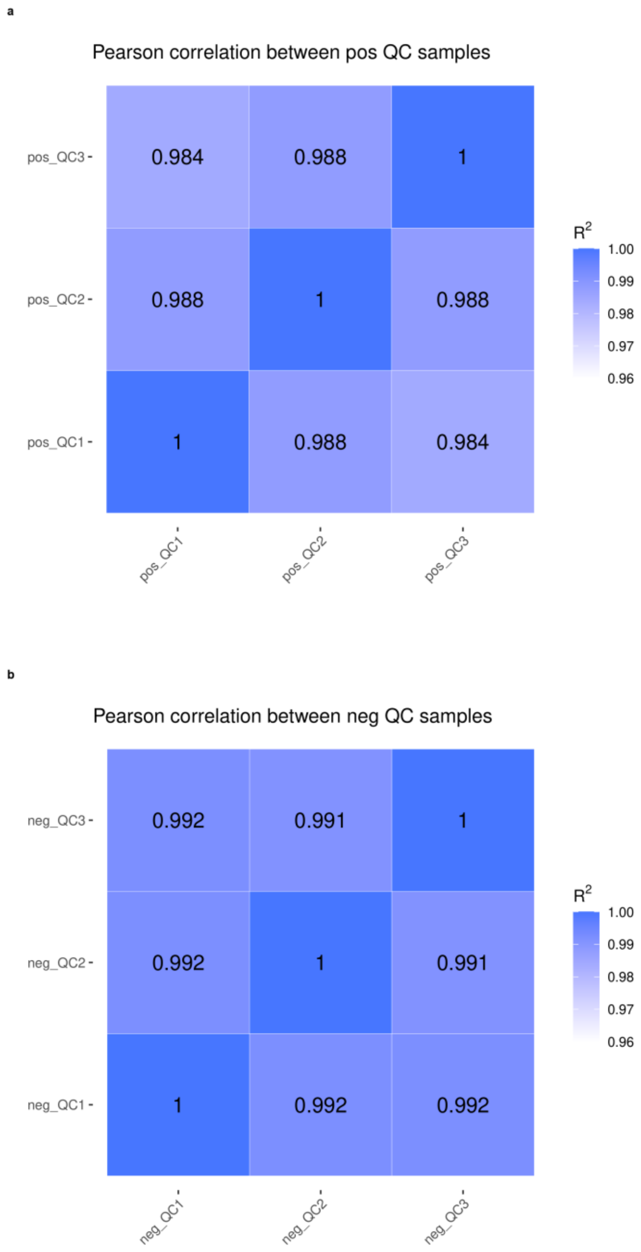

Figure S1 Pearson correlation coefficient diagram of QC samples (a. positive ion mode; b. negative ion mode)

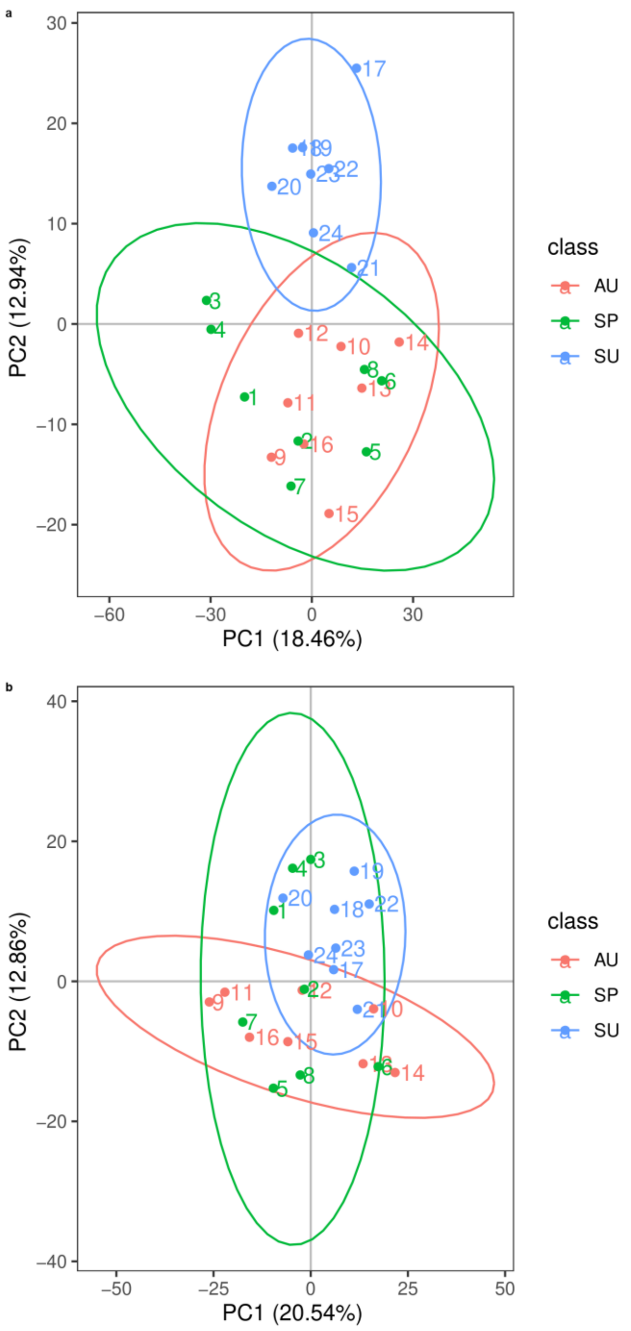

Figure S2 PCA analysis diagram of metabolites (a. positive ion mode; b. negative ion mode)

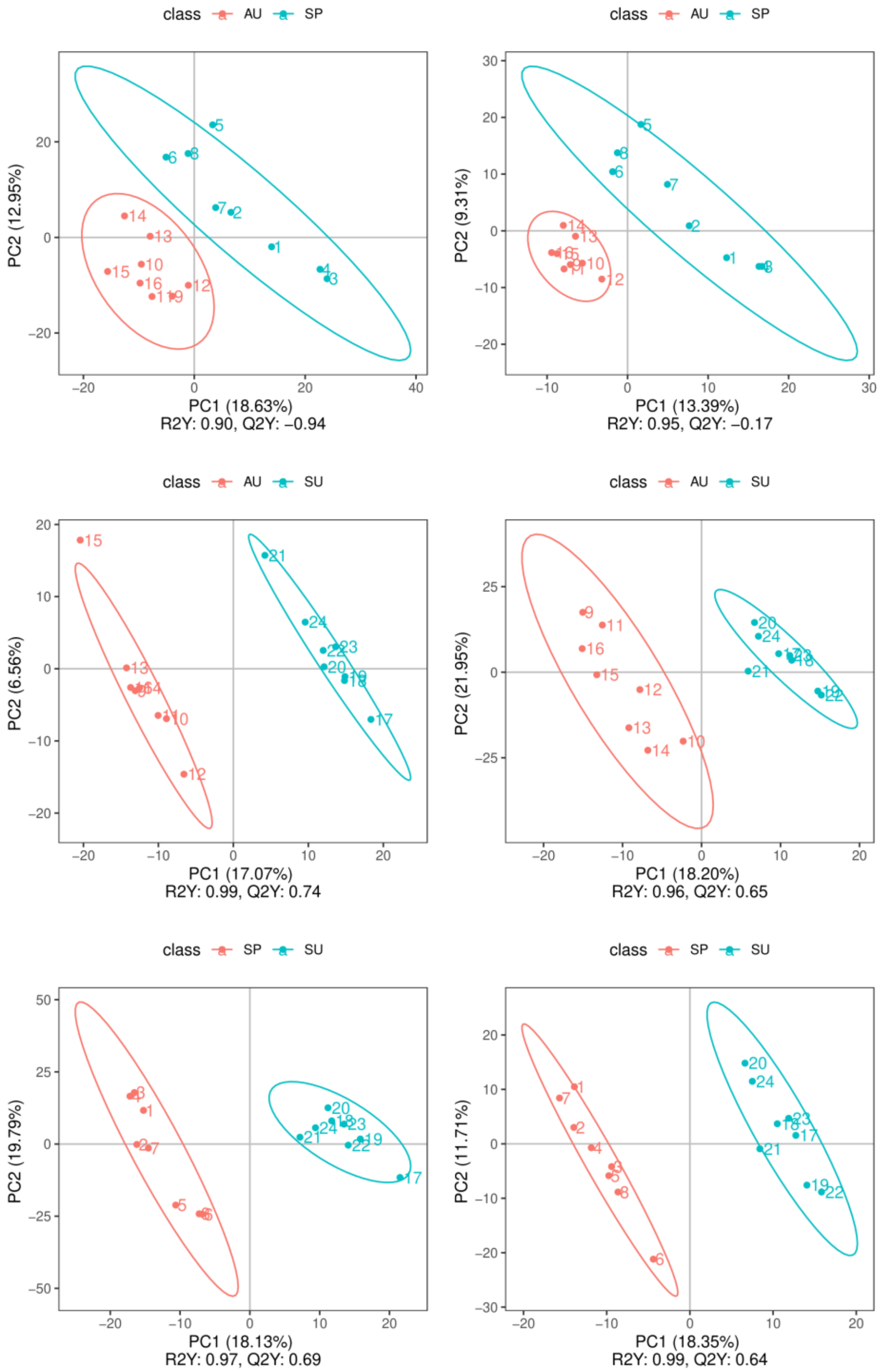

Figure S3 PLS-DA analysis results among different groups (positive ion mode on the left, negative ion mode on the right)

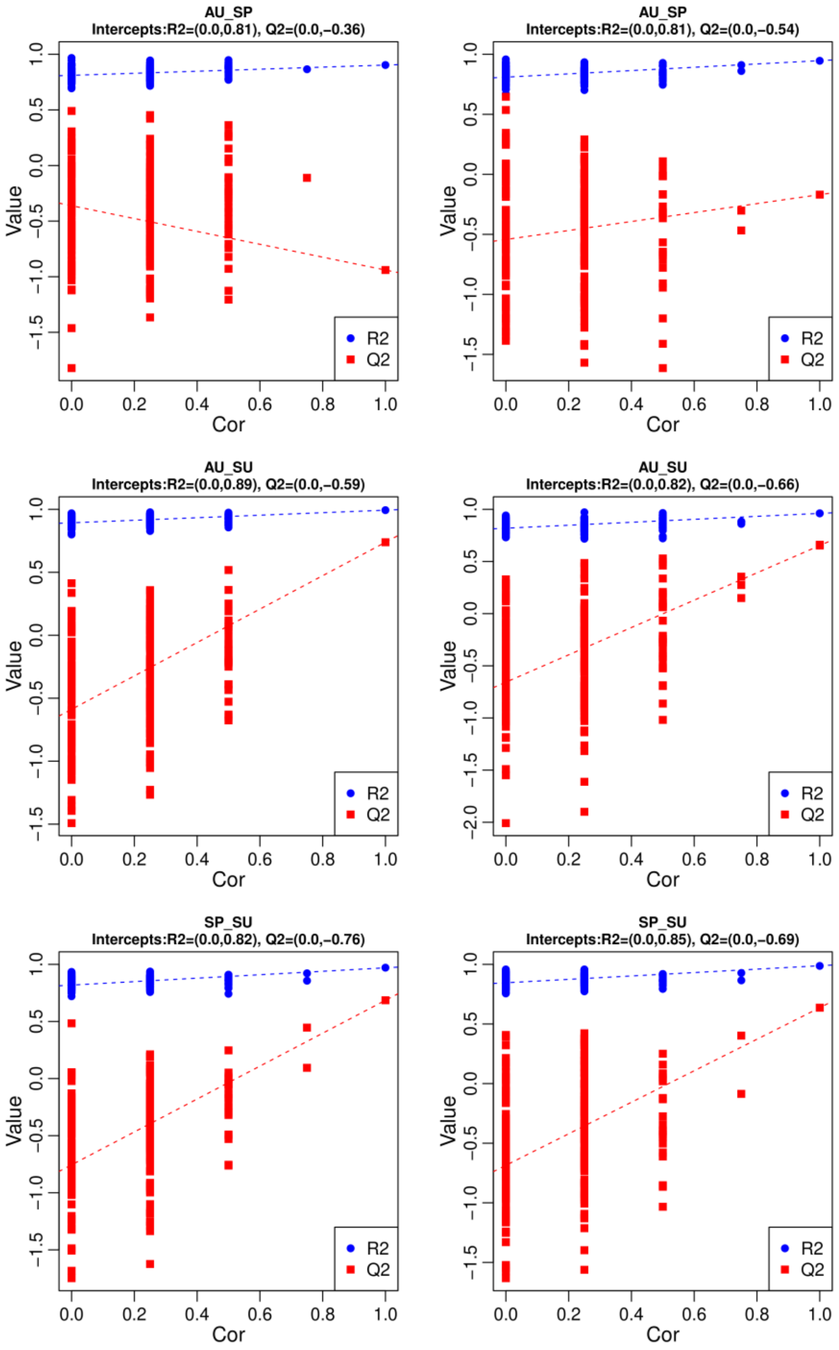

Figure S4 200-permutation test results of PLS-DA models among different groups (positive ion mode on the left, negative ion mode on the right)

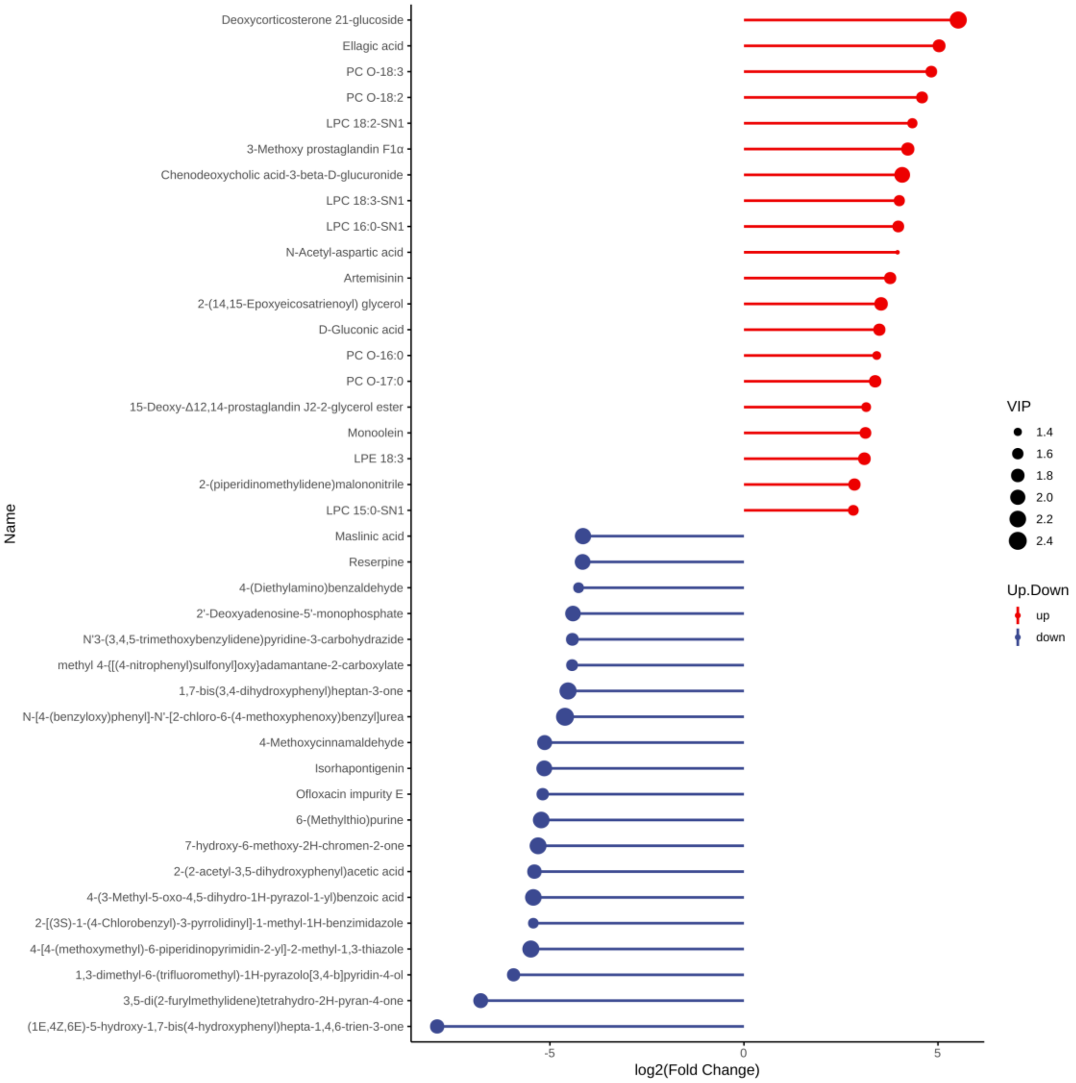

Figure S5 Matchstick diagram of differential metabolites between SP and SU groups (positive ion mode)

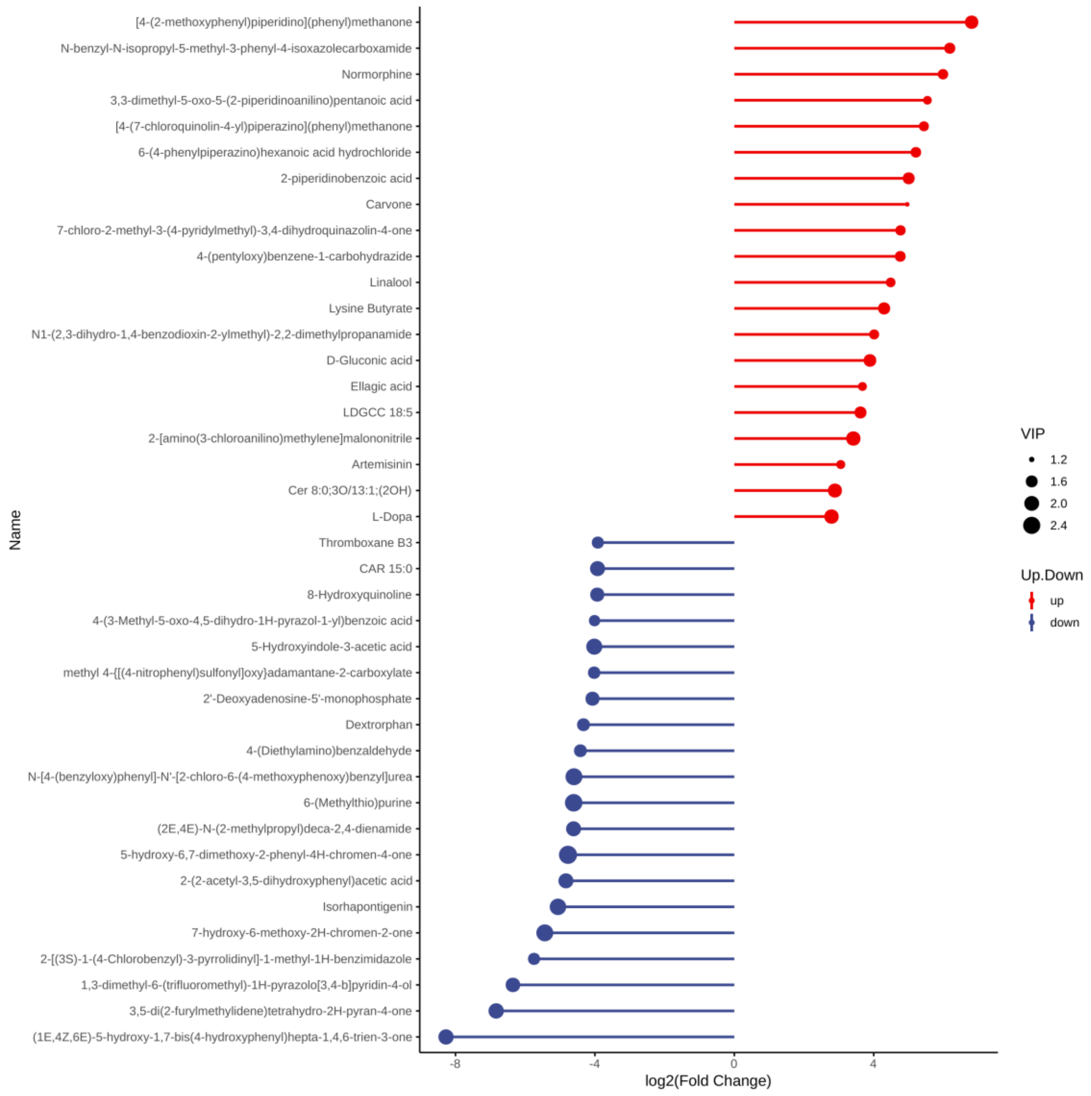

Figure S6 Matchstick diagram of differential metabolites between AU and SU groups (positive ion mode)

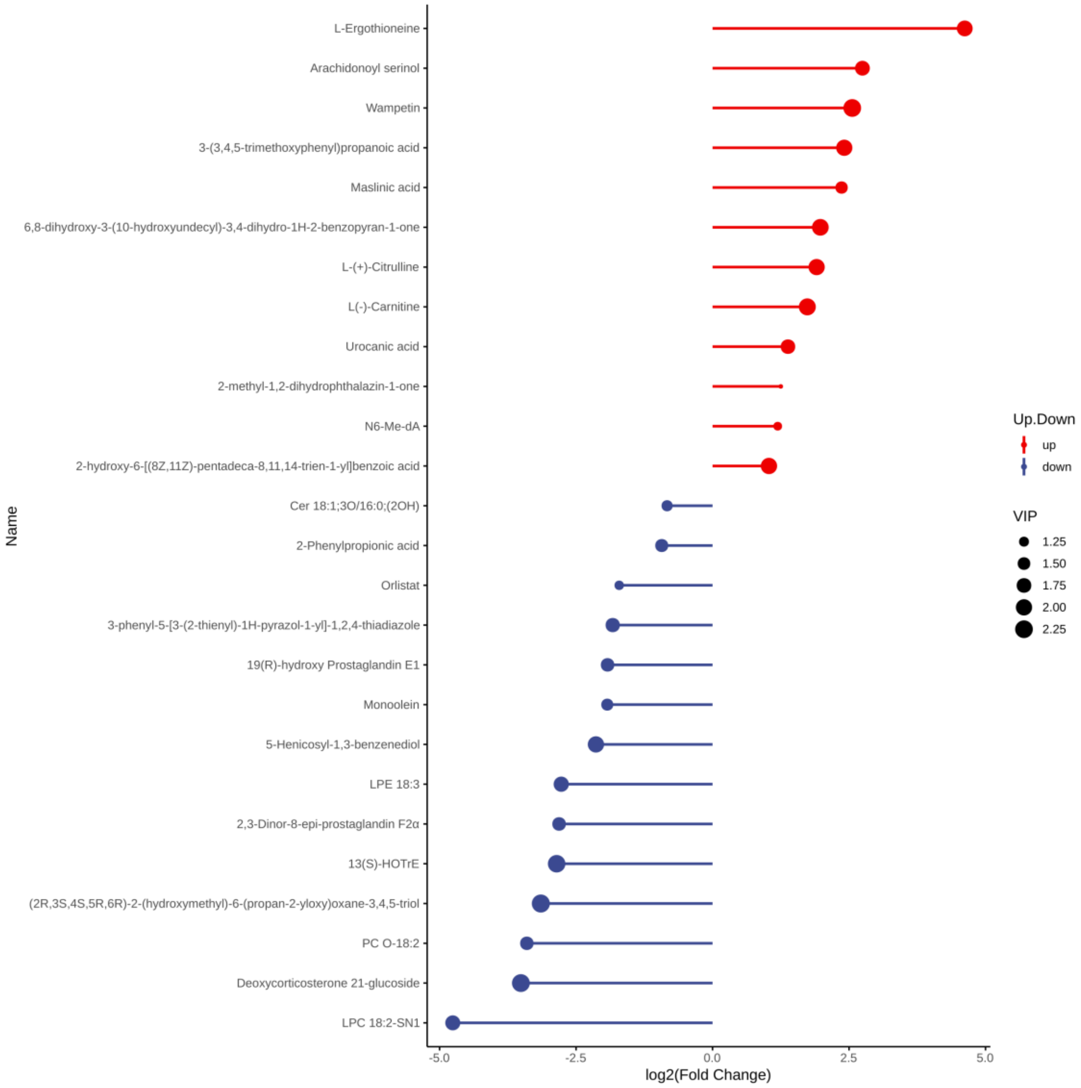

Figure S7 Matchstick diagram of differential metabolites between AU and SP groups (positive ion mode)

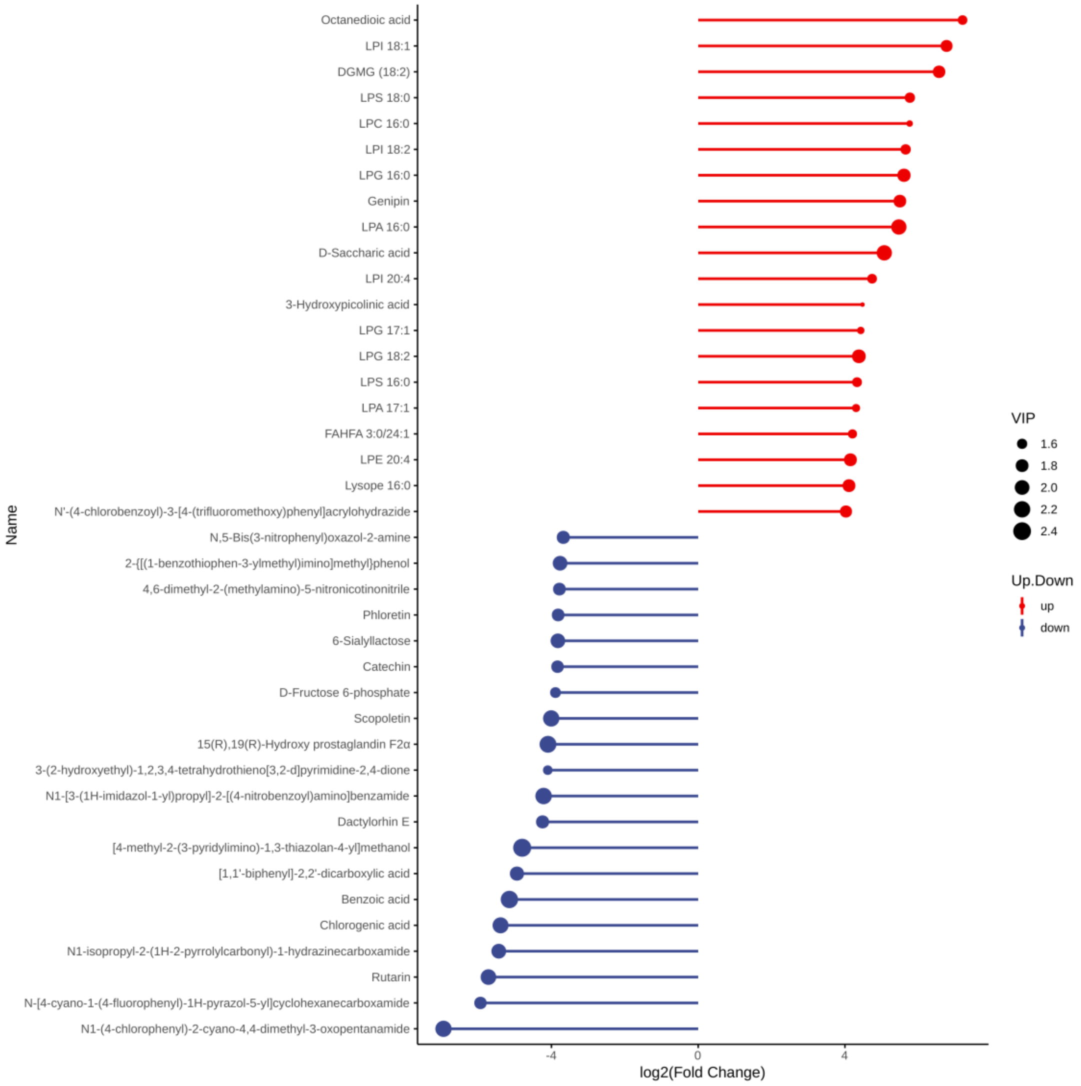

Figure S8 Matchstick diagram of differential metabolites between SP and SU groups (negative ion mode)

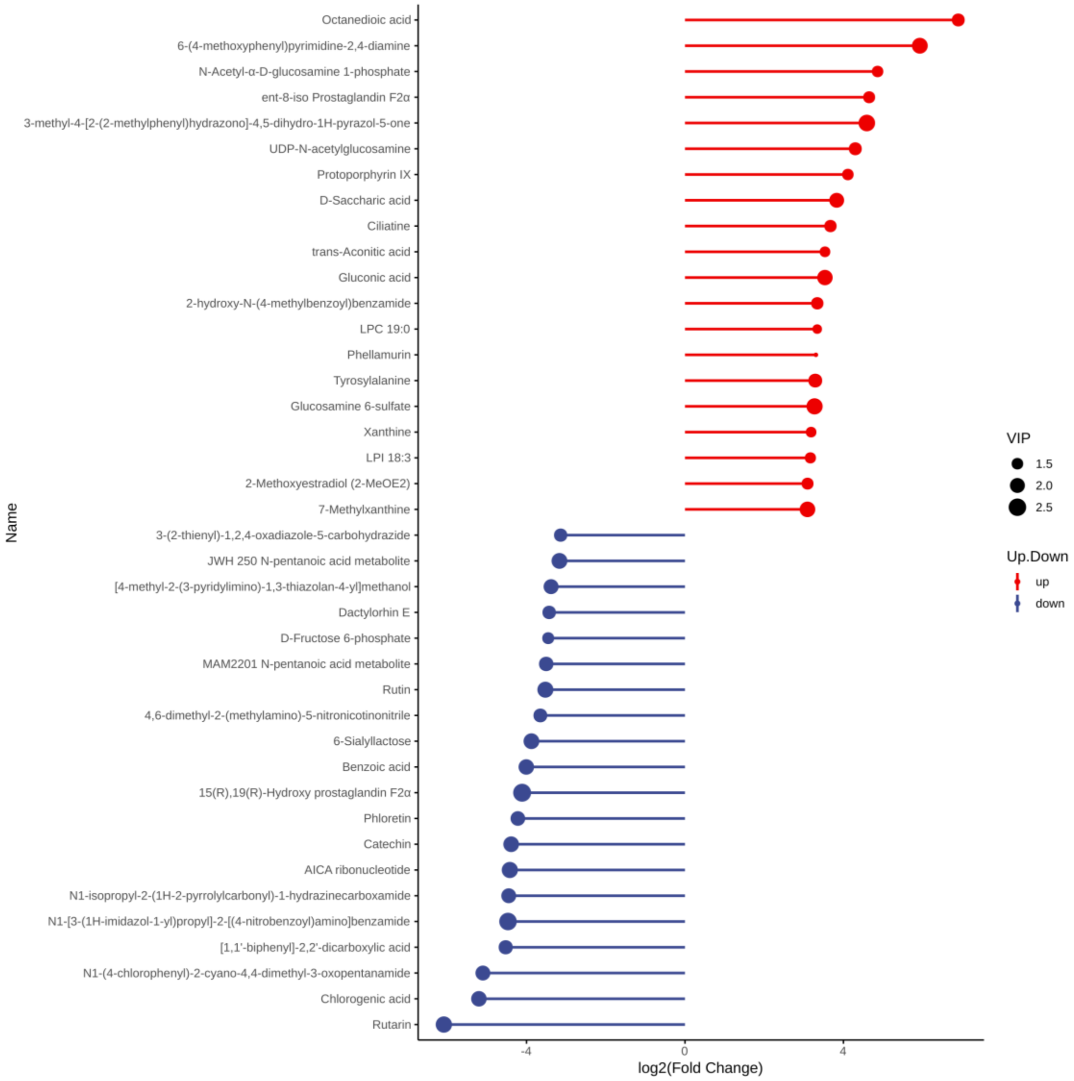

Figure S9 Matchstick diagram of differential metabolites between AU and SU groups (negative ion mode)

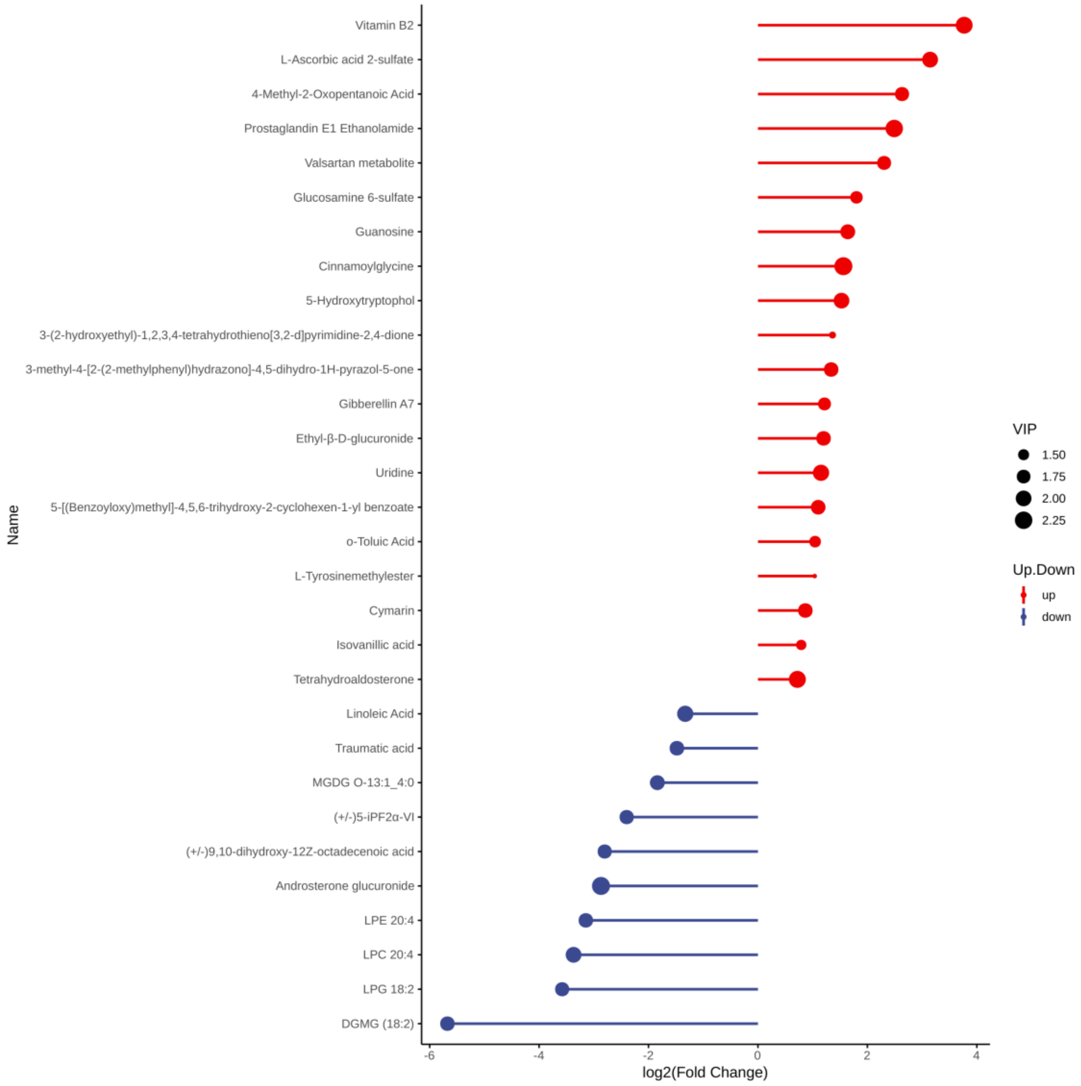

Figure S10 Matchstick diagram of differential metabolites between AU and SP groups (negative ion mode)

a

b

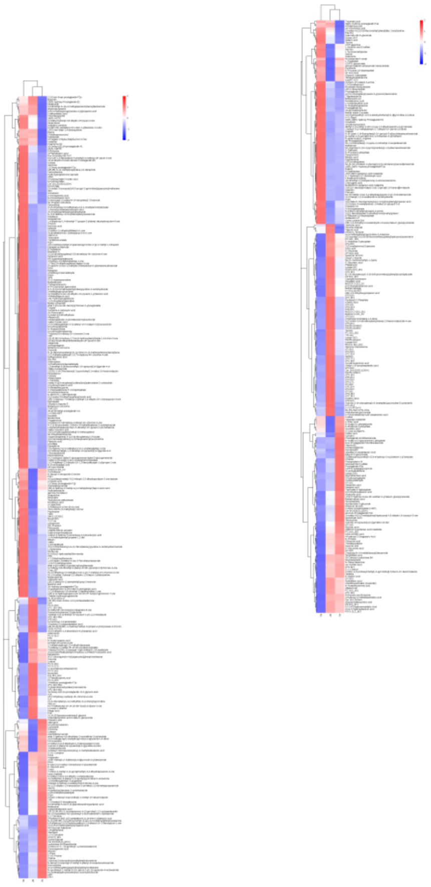

Figure S11 Clustering heat map of total differential metabolites (a. positive ion mode; b. negative ion mode)

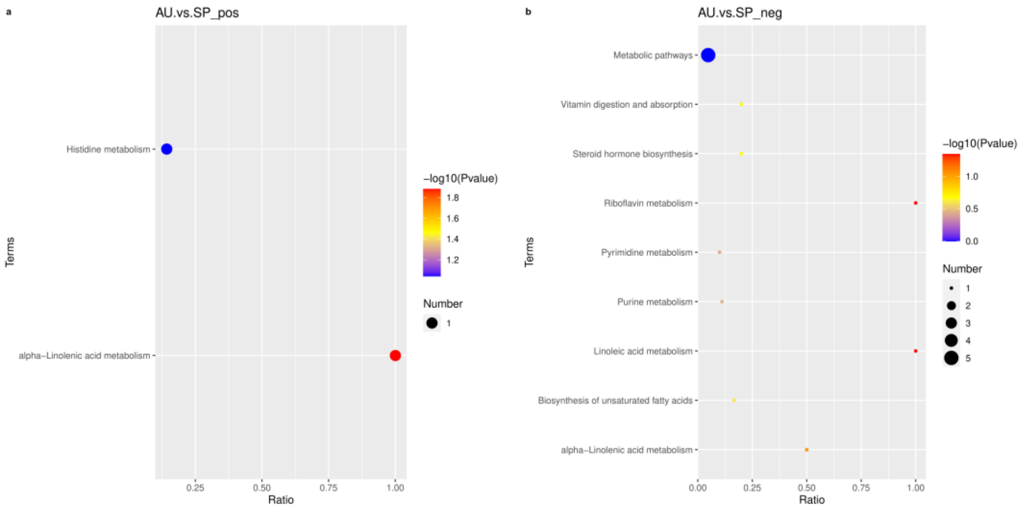

Figure S12 Enrichment results of KEGG pathway between AU and SP groups (a. positive ion mode; b. negative ion mode)

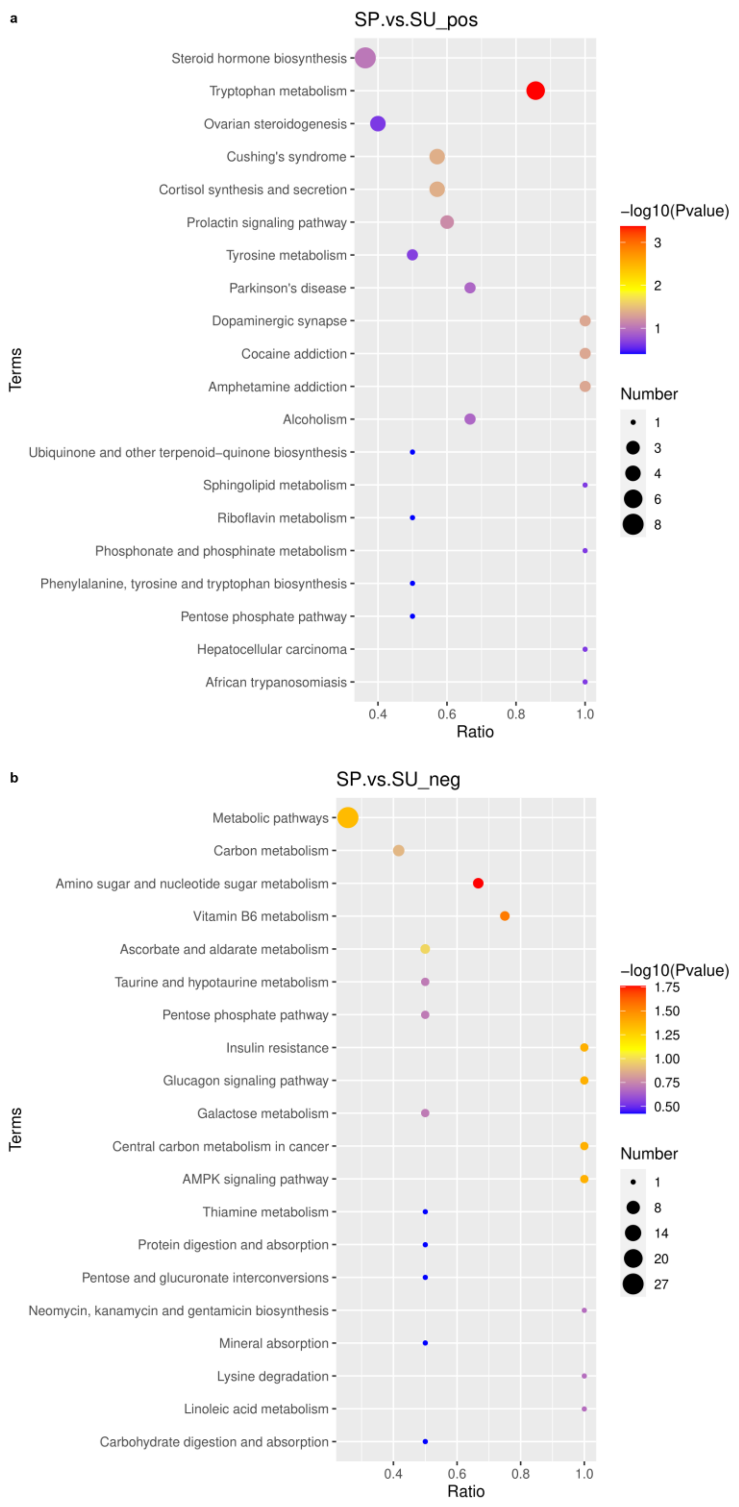

Figure S13 Enrichment results of KEGG pathway between SP and SU groups (a. positive ion mode; b. negative ion mode)

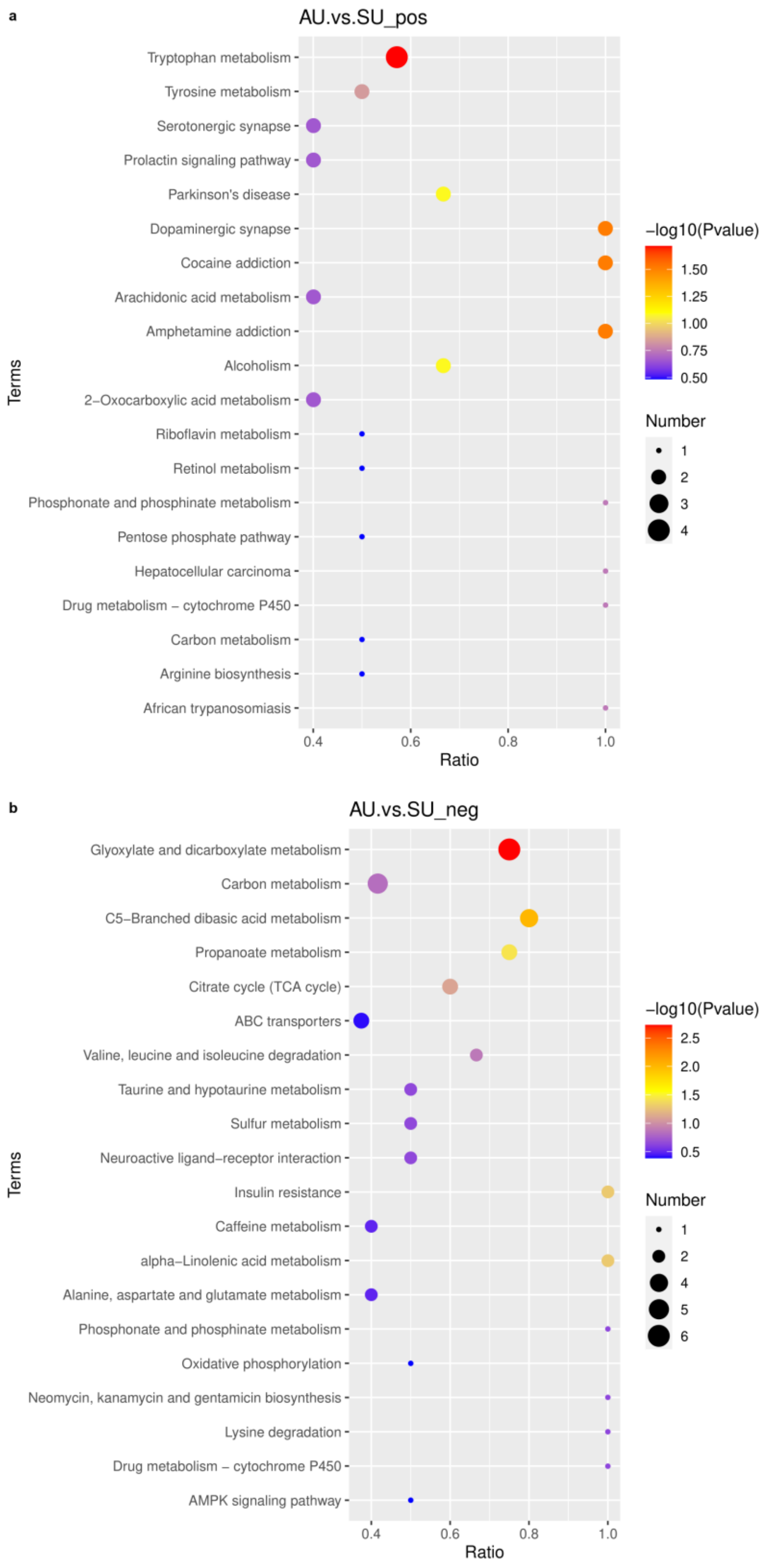

Figure S14 Enrichment results of KEGG pathway between AU and SU groups (a. positive ion mode; b. negative ion mode)
